# Supplementary material for: Modelling the first wave of COVID-19 in India
Source: PLoS Comput Biol. 2022 Oct 24;18(10):e1010632. doi: 10.1371/journal.pcbi.1010632 (PMC9632871; doi:10.1371/journal.pcbi.1010632)
Supplement: S1 Text — Section 1: Computation of reproduction number R0. We provide the calculation of the basic reproductive ratio corresponding to the age-stratified model, assuming uniform infectivity and values of ϵ = 1, using a next-generation-matrix method. Section 2: Contact matrices between stratified age-groups. We describe how the contact matrices of Ref. [75] can be specified for our use here, combining contact matrices with a 5-year resolution into a more coarse-grained description. Fig A: The coarse-grained contact matrices for India during (top) and without lockdown (bottom). Section 3: Brief discussion on Nested Sampling. We briefly discuss the sampling method we have used in our analysis. Section 4: Flowchart of our analysis. We discuss the essentials of the algorithm and techniques and provide a flowchart of the analysis. Fig B: A schematic flowchart of our analysis. Section 5: Supporting plots for Karnataka, Mumbai and India (aggregate) analysis. We present supporting plots and tables corresponding to our analysis for Karnataka state, Mumbai and for India-wide numbers. Table A: Karnataka: Constraints on parameters. Fig C: Karnataka: Bounds on cumulative infection with no death multiplier. Fig D: Karnataka: Bounds on cumulative infection with a death multiplier of 2.2. Fig E: Karnataka: Bounds on cumulative infection with a death multiplier of 5. Fig F: Karnataka: Marginalized posteriors of the parameters. Table B: Mumbai: Constraints on parameters. Fig G: Mumbai: Bounds on cumulative infection with no death multiplier. Fig H: Mumbai: Marginalized posteriors of the parameters. Table C: India: Mean and 95% bounds on the parameters. Fig I: India: Bounds on cumulative infection with a death multiplier of 2.2. Fig J: India: Marginalized posteriors of the parameters. Section 6: Infection curves for select Indian cities. In the main text, we presented the analysis of Mumbai alone. Here, following the same protocol, we describe results for Bengaluru Urban, Chennai, Pune, and Del [file pcbi.1010632.s001.pdf]

Supplementary materials for article: Modelling the first wave of COVID-19 in India

Dhiraj Kumar Hazra, Bhalchandra S. Pujari, Snehal M. Shekatkar, Farhina Mozaffer, Sitabhra Sinha, Vishwesha Guttal, Pinaki Chaudhuri, Gautam I. Menon

October 19, 2022

Contents

1 Computation of reproduction number R_0 3

2 Contact matrices between stratified age-groups 4

3 Brief discussion on Nested Sampling 4

4 Flowchart of our analysis 4

5 Supporting plots for Karnataka, Mumbai and India (aggregate) analysis 8
- 5.1 Posterior distributions and correlations between parameters . . . 8
- 5.2 Plots for cumulative infection and deaths . . . 8
- 5.3 Karnataka . . . 8
- 5.4 Mumbai . . . 11
- 5.5 India . . . 13

6 Infection curves for select Indian cities 15
- 6.1 Bengaluru Urban . . . 15
- 6.2 Chennai . . . 18
- 6.3 Delhi . . . 21
- 6.4 Pune . . . 24

List of Figures

A The coarse-grained contact matrices for India without lockdown (top) and during lockdown (bottom) . . . 5

B Schematic diagram of our analysis. . . . 7

C Karnataka: Bounds on cumulative infection [a: left] and deaths [b: right] from our analysis plotted with reported data. . . . 9

D Karnataka: Bounds on cumulative infection [a: left] and deaths [b: right] from our analysis plotted with reported data. Note that here death multiplier 2.2 is used to take into account possible death undercounting. . . . 9

E Karnataka: Bounds on cumulative infection [a: left] and deaths [b: right] from our analysis plotted with reported data. Note that here death multiplier 5 is used to take into account possible death undercounting. . . . 9

F Karnataka: Marginalized posteriors of the parameters in the adaptive parametrization in the INDSCI-SIM model against the Karnataka data. We have plotted the constraints obtained assuming and without assuming death multiplier for undercounting. We use death multipliers of 1, 2.2 and 5 to consider the following scenarios – no death undercounting, average and high undercounting obtained from districts. Please refer to Table 3 in the main text and Section 1.8 and 1.9 in the main text for detailed descriptions of the symbols. . . . 10

G Mumbai: Bounds on cumulative infection [a: left] and deaths [b: right] from our analysis plotted with reported data. . . . 11

H Mumbai: Marginalized posteriors of the parameters in the adaptive parametrization in the INDSCI-SIM model against the Mumbai data. Please refer to Table 3 in the main text and Section 1.8 and 1.9 in the main text for detailed descriptions of the symbols. . . . 12

I India: Bounds on cumulative infection [a: left] and deaths [b: right] from our analysis plotted with reported data. Note that here death multiplier 2.2 is used to take into account possible death undercounting. . . . 13

J India: Marginalized posteriors of the parameters in the adaptive parametrization for India-level data. Please refer to Table 3 in the main text and Section 1.8 and 1.9 in the main text for detailed descriptions of the symbols. . . . 14

K Bengaluru Urban: Timeseries analysis for Bengaluru Urban. We plot the fit to the daily infected cases [a: top left] and daily reported deaths [b: top right] assuming no death undercounting. The middle panel contains the cumulative actual infected cases [c: left] and the bias multiplicative factor [d: right] obtained as a ratio between actual and reported infections. The left plot [e] at the bottom panel contains the evolution of the age averaged IFR. The bottom right plot [f] contains our estimation of $R(t)$ and an independent [1] measurement. Note that the bands correspond to 2σ and 3σ confidence levels. . . . 16
L Bengaluru Urban: Bounds on cumulative infection [a: left] and deaths [b: right] from our analysis plotted with reported data. . . . 16
M Bengaluru Urban: Marginalized posteriors of the parameters in the adaptive parametrization in the INDSCI-SIM model against the Bengaluru Urban data. Please refer to Table 3 in the main text and Section 1.8 and 1.9 in the main text for detailed descriptions of the symbols. 17
N Chennai: Timeseries analysis for Chennai. We plot the fit to the daily infected cases [a: top left] and daily reported deaths [b: top right] assuming no death undercounting. Middle panel contains the cumulative actual infected cases [c: left] and the bias multiplicative factor [d: right] obtained as a ratio between actual and reported infections. The left plot [e] at the bottom panel contains the evolution of age averaged IFR. The bottom right plot [f] contains our estimation of $R(t)$ and an independent [1] measurement. Note that the bands correspond to 2σ and 3σ confidence levels. . . . 19
O Chennai: Bounds on cumulative infection [a: left] and deaths [b: right] from our analysis plotted with reported data. . . . 19
P Chennai: Marginalized posteriors of the parameters in the adaptive parametrization in the INDSCI-SIM model against the Chennai data. Please refer to Table 3 in the main text and Section 1.8 and 1.9 in the main text for detailed descriptions of the symbols. . . . 20
Q Delhi: Timeseries analysis for Delhi. We plot the fit to the daily infected cases [a: top left] and daily reported deaths [b: top right] assuming no death undercounting. Middle panel contains the cumulative actual infected cases [c: left] and the bias multiplicative factor [d: right] obtained as a ratio between actual and reported infections. The left plot [e] at the bottom panel contains the evolution of age averaged IFR. The bottom right plot [f] contains our estimation of $R(t)$ and an independent [1] measurement. Note that the bands correspond to 2σ and 3σ confidence levels. . . . 21
R Delhi: Bounds on cumulative infection [a: left] and deaths [b: right] from our analysis plotted with reported data. . . . 22
S Delhi: Marginalized posteriors of the parameters in the adaptive parametrization in the INDSCI-SIM model against the Delhi data. Please refer to Table 3 in the main text and Section 1.8 and 1.9 in the main text for detailed descriptions of the symbols. . . . 23
T Pune: Timeseries analysis for Pune. We plot the fit to the daily infected cases [a: top left] and daily reported deaths [b: top right] assuming no death undercounting. Middle panel contains the cumulative actual infected cases [c: left] and the bias multiplicative factor [d: right] obtained as a ratio between actual and reported infections. The left plot [e] at the bottom panel contains the evolution of age averaged IFR. The bottom right plot [f] contains our estimation of $R(t)$ and an independent [1] measurement. Note that the bands correspond to 2σ and 3σ confidence levels. . . . 25
U Pune: Bounds on cumulative infection [a: left] and deaths [b: right] from our analysis plotted with reported data. . . . 25
V Pune: Marginalized posteriors of the parameters in the adaptive parametrization in the INDSCI-SIM model against the Pune data. Please refer to Table 3 in the main text and Section 1.8 and 1.9 in the main text for detailed descriptions of the symbols. . . . 26

List of Tables

A Karnataka: Constraints on parameters. Corresponding to Fig F the 95% constraints and bounds are provided. Two columns on constraints represent the results when we use reported death data without and with undercounting multiplier 2.2 and 5 respectively. Please refer to Table 3 in the main text and Section 1.8 and 1.9 in the main text for detailed descriptions of the symbols. . . . 8
B Mumbai: Constraints on parameters. Corresponding to Fig H the 95% constraints and bounds are provided. Please refer to Table 3 in the main text and Section 1.8 and 1.9 in the main text for detailed descriptions of the symbols. . . . 11
C India: Mean and 95% bounds on the parameters. These values correspond to the posteriors plotted in Fig J. Please refer to Table 3 in the main text and Section 1.8 and 1.9 in the main text for detailed descriptions of the symbols. . . . 13
D Bengaluru Urban: Constraints on parameters. Corresponding to Fig M the 95% constraints and bounds are provided. Please refer to Table 3 in the main text and Section 1.8 and 1.9 in the main text for detailed descriptions of the symbols. . . . 15
E Chennai: Constraints on parameters. Corresponding to Fig P the 95% constraints and bounds are provided. Please refer to Table 3 in the main text and Section 1.8 and 1.9 in the main text for detailed descriptions of the symbols. . . . 18
F Delhi: Constraints on parameters. Corresponding to Fig S the 95% constraints and bounds are provided. Please refer to Table 3 in the main text and Section 1.8 and 1.9 in the main text for detailed descriptions of the symbols. 22
G Pune: Constraints on parameters. Corresponding to Fig V the 95% constraints and bounds are provided. Please refer to Table 3 in the main text and Section 1.8 and 1.9 in the main text for detailed descriptions of the symbols. 24

1 Computation of reproduction number R_0

Assuming uniform contacts across all age groups using the infectivity parameter β and efficiency parameters ϵ_i, the $\mathcal{F}$ matrix [2, 3] can be written as:

$$\mathcal{F} = \begin{pmatrix} 0 & \beta\epsilon_a(t) & \beta\epsilon_p(t) & \beta\epsilon_m(t) & \beta\epsilon_s(t) \\ 0 & 0 & 0 & 0 & 0 \\ 0 & 0 & 0 & 0 & 0 \\ 0 & 0 & 0 & 0 & 0 \\ 0 & 0 & 0 & 0 & 0 \end{pmatrix} \tag{1}$$

Here a, p, m, s correspond to asymptomatic, pre-symptomatic, mild and severe compartments respectively.
And the $\mathcal{V}$ matrix is obtained as,

$$\mathcal{V} = \begin{pmatrix} \gamma & 0 & 0 & 0 & 0 \\ -\alpha\gamma & \lambda_a & 0 & 0 & 0 \\ -1(1-\alpha)\gamma & 0 & \lambda_p & 0 & 0 \\ 0 & 0 & -\mu\lambda_p & \lambda_m & 0 \\ 0 & 0 & -(1-\mu)\lambda_p & 0 & \lambda_s \end{pmatrix} \tag{2}$$

Here we have used the fractions and rates as defined in the main article.

$$\mathcal{V}^{-1} = \begin{pmatrix} \frac{1}{\gamma} & 0 & 0 & 0 & 0 \\ \frac{\alpha}{\lambda_a} & \frac{1}{\lambda_a} & 0 & 0 & 0 \\ \frac{1-\alpha}{\lambda_p} & 0 & \frac{1}{\lambda_p} & 0 & 0 \\ \frac{\mu-\alpha\mu}{\lambda_m} & 0 & \frac{\mu}{\lambda_m} & \frac{1}{\lambda_m} & 0 \\ \frac{(\alpha-1)(\mu-1)}{\lambda_s} & 0 & \frac{1-\mu}{\lambda_s} & 0 & \frac{1}{\lambda_s} \end{pmatrix} \tag{3}$$

The next generation matrix:

$$\mathcal{F}\mathcal{V}^{-1} = \begin{pmatrix} \beta\left(\frac{\alpha\epsilon_a(t)}{\lambda_a} - \frac{(\alpha-1)\mu\epsilon_m(t)}{\lambda_m} - \frac{(\alpha-1)\epsilon_p(t)}{\lambda_p} + \frac{(\alpha-1)(\mu-1)\epsilon_s(t)}{\lambda_s}\right) & \frac{\beta\epsilon_a(t)}{\lambda_a} & \beta\left(\frac{\mu\epsilon_m(t)}{\lambda_m} + \frac{\epsilon_p(t)}{\lambda_p} - \frac{(\mu-1)\epsilon_s(t)}{\lambda_s}\right) & \frac{\beta\epsilon_m(t)}{\lambda_m} & \frac{\beta\epsilon_s(t)}{\lambda_s} \\ 0 & 0 & 0 & 0 & 0 \\ 0 & 0 & 0 & 0 & 0 \\ 0 & 0 & 0 & 0 & 0 \\ 0 & 0 & 0 & 0 & 0 \end{pmatrix} \tag{4}$$

The dominant eigenvalue of the next generation matrix is R_0.

$$R_0 = \beta\left(\frac{\alpha\epsilon_a(t)}{\lambda_a} + (\alpha-1)\left(-\frac{\mu\epsilon_m(t)}{\lambda_m} - \frac{\epsilon_p(t)}{\lambda_p} + \frac{(\mu-1)\epsilon_s(t)}{\lambda_s}\right)\right) \tag{5}$$

Since INDSCI-SIM model is age stratified and uses contact matrices, the $\mathcal{F}$ and $\mathcal{V}$ matrices are generalized according to the model. Given N age groups and M equations determining the Exposed and infected compartments we will have $\mathcal{F}$ and $\mathcal{V}$ matrices of order $NM \times NM$. In our model we have 9 age compartments and 5 equations for exposed and 4 infected compartments. Therefore the $\mathcal{F}$ and $\mathcal{V}$ are of order 45×45.

The modified $\mathcal{F}$ matrix can be written as:

$$\mathcal{F} = \begin{pmatrix} [0] & [\mathcal{B}_i(t)] & \dots \\ \vdots & \ddots & \\ [0] & \dots & [0] \end{pmatrix} \tag{6}$$

Here $[0]$ represents $N \times N$ matrix with all entries as 0. $[\mathcal{B}_i(t)]$ represents the time dependent effective infectivity matrix (order $N \times N$) for different infected compartments and is defined as follows:

$$[\mathcal{B}_i(t)] = \epsilon_i\tilde{\beta}\exp[-t/\tau_i][C], \tag{7}$$

where the m, n'th element of the matix $[C]$ are defined with the contact matrix C_{mn} and the population fraction f_m as $C_{mn}f_m/f_n$. Here τ_i parameter represents the characteristic time-scale describing the increased effectiveness of non-pharmaceutical interventions, as introduced in the main paper. Note that β in the unifrom contact case and $\tilde{\beta}$ in the differential contacts are different. In the uniform contact β represents the infectivity averaged over all contacts while in this case $\tilde{\beta}$ represents the infectivity per contact. Note that the contact matrix changes during lockdown and therefore changes the effective infectivity matrix.

Generalized $\mathcal{V}$ matrix is now denoted as,

$$\mathcal{V} = \begin{pmatrix} [\Gamma] & [0] & [0] & [0] & [0] \\ -[A\Gamma] & [\Lambda_a] & [0] & [0] & [0] \\ -[(\mathbb{1}-A)\Gamma] & [0] & [\Lambda_p] & [0] & [0] \\ [0] & [0] & -[M\Lambda_p] & [\Lambda_m] & [0] \\ [0] & [0] & -[(\mathbb{1}-M)\Lambda_p] & [0] & [\Lambda_s] \end{pmatrix} \tag{8}$$

In the above $NM \times NM$ matrix each entry in square brackets represents a $N \times N$ matrix. In our model $M = 5$. For models with more infected compartments, M will increase for both $\mathcal{F}$ and $\mathcal{V}$ matrices. Γ, Λ_i's represent diagonal matrices with the transition rates as the diagonal terms. In our case, we have assumed the rates of transfer between compartments are same for all age groups. In a more general model, where the rates are different, the diagonal entries will change. The asymptomatic fractions and mild fractions are also represented by diagonal matrices, A and M respectively. Note that our fractions are age dependent and therefore, the diagonal terms contain the fractions for 9 age groups.

With the $\mathcal{F}$ and $\mathcal{V}$ matrices prepared, we follow the same procedure as in the uniform contact case and the dominated eigenvalue of $\mathcal{F}\mathcal{V}^{-1}$ is estimated as R_0.

At any point in time, while the basic reproduction number R_0 can be obtained using the relations above, the effective reproduction number $R(t)$ takes into account the susceptible population remaining at time t *w.r.t.* the initial susceptible population. Thus, $R(t) = S(t = t)R_0/S(t = 0)$ where the susceptible population at $t = 0$ and $t = t$ are given by $S(t = 0)$ and $S(t = 0)$ respectively.

2 Contact matrices between stratified age-groups

Mixing between different age groups is determined by age-specific contact matrices. To compute these, we use contact matrices provided by [4], estimated for the Indian population, for 16 age groups ranging between 0-80 years, tabulated at 5 year intervals.

We reduce the matrix to further coarse-grained age brackets as follows: Let the number of contacts of the i-th individual in the age category j with any individual belonging to the age category k be $C_{kj}(i)$. The mean number of contacts for age category j with age category k is thus $\bar{C}_{kj} = \sum_{i=1}^{N_j} C_{kj}(i)/N_j$, where N_j is the population size in age category j. Suppose now that k is subdivided into 2 finer categories k_1 and k_2, and we are given the entries $\bar{C}_{k_1k_1}$, $\bar{C}_{k_1k_2}$, $\bar{C}_{k_2k_1}$ and $\bar{C}_{k_2k_2}$, as well as, $\bar{C}_{jk_1}$, $\bar{C}_{jk_2}$, $\bar{C}_{k_1j}$ and $\bar{C}_{k_2j}$. We then have

$$C_{kk} = [N_{k_1}\bar{C}_{k_1k_1} + N_{k_1}\bar{C}_{k_2k_1} + N_{k_2}\bar{C}_{k_1k_2} + N_{k_2}\bar{C}_{k_2k_2}]/(N_{k_1} + N_{k_2}) \tag{9}$$

with the off diagonal terms defined as $C_{jk} = [N_{k_1}\bar{C}_{jk_1} + N_{k_2}\bar{C}_{jk_2}]/(N_{k_1} + N_{k_2})$, and $C_{kj} = \bar{C}_{k_1j} + \bar{C}_{k_2j}$. We use this reduced matrix $[C]$ in our analysis. We plot the contact matrices in Fig A.

3 Brief discussion on Nested Sampling

Since the posterior probabilities are expected to be multi-modal Markov Chain Monte Carlo can not be used. Therefore we use nested sampling with `PolyChord` that is effective for higher dimensional parameter spaces [5, 6]. This algorithm uses live points that are updated in each iteration shrinking the n-dimensional parameter spaces. We use 500-1000 live points according to the dimension of the parameter space for the model. `Polychord` starts with the live points and the points are sequentially updated in each iteration. Point with the lowest live point is discarded (termed as dead point) and replaced by a new point with a likelihood higher than that of the dead point. It can be shown [5] that the prior volume shrinks exponentially with each iteration. The evidence or the marginal likelihood is computed from the integral of likelihood and prior over the prior volume.

4 Flowchart of our analysis

In Fig B we plot the logical flow of the analysis. Centrally, we use 2 codes. For simulation, we have developed `ELiXSIR` – *Extended, zone Linked IX-compartmental SIR model: a code to simulate COVID19 infection* [7]. For sampling we use `PolyChord` [5], a widely used code for nested sampling. Both codes are available publicly.

Given a set of parameters we set up the system for `ELiXSIR`. In this set-up we provide the population, number of age groups and fraction of population in these age groups. We fix the fraction and rates of transition between compartments that are runtime constants. Lockdown dates are mentioned based on which the code switches from lockdown to unlock modes switching contact matrices. We use the coarse grained contact matrices for each region using the population fractions within the age groups of base contact matrix and the coarse grained age groups [4].

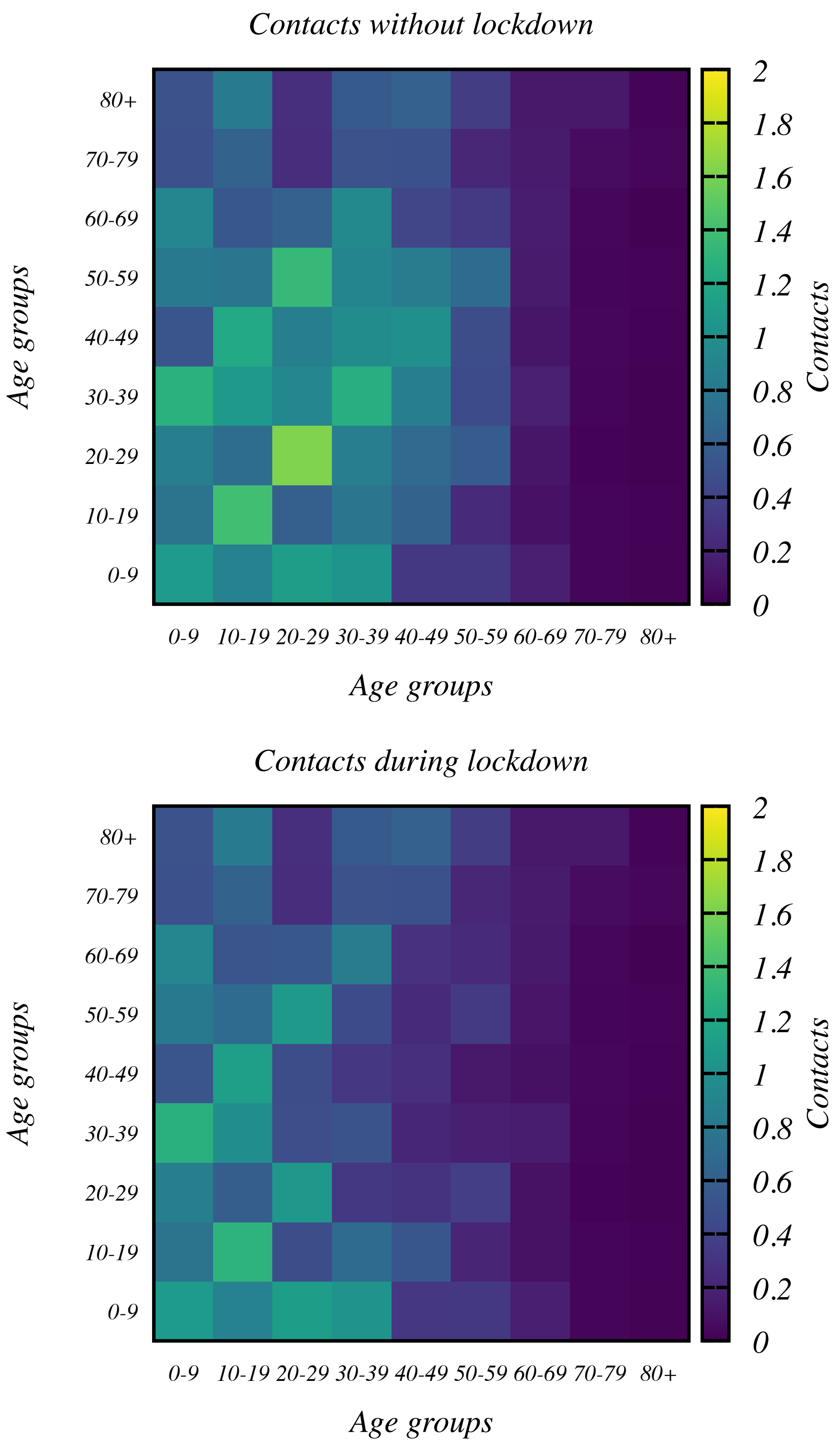

Figure A: The coarse-grained contact matrices for India without lockdown (top) and during lockdown (bottom)

.

After the initial set up, the priors, initial starting value of all parameter and widths are provided. Daily data of reported infection and deaths are supplied to the code with the dates.

The CosmoChord integrated with ELiXSIR is then run in several processors (MPI) in the cluster. The samples within the parameter volume are drawn and sent to ELiXSIR for the time-series. Daily infection and deaths are computed from the compartments. Bias evolution is generated according to the parametric form and the values of b, Δ_b from the samples. After scaling the theoretically obtained daily infection numbers by the bias, the prediction of daily infected is then compared with the reported daily infection. Prediction of daily deaths however is directly compared with the daily deaths data. If the death undercounting factor is included in the analysis then the daily deaths prediction is compared with the death data scaled by the undercounting factor. During the runtime, live points are generated and updated sequentially that reduces the prior volume, as discussed in the last section.

After termination of the PolyChord sampling, we use getdist [8] to generate posterior distributions. The samples are then supplied to ELiXSIR directly to generate the bounds on the timeseries.

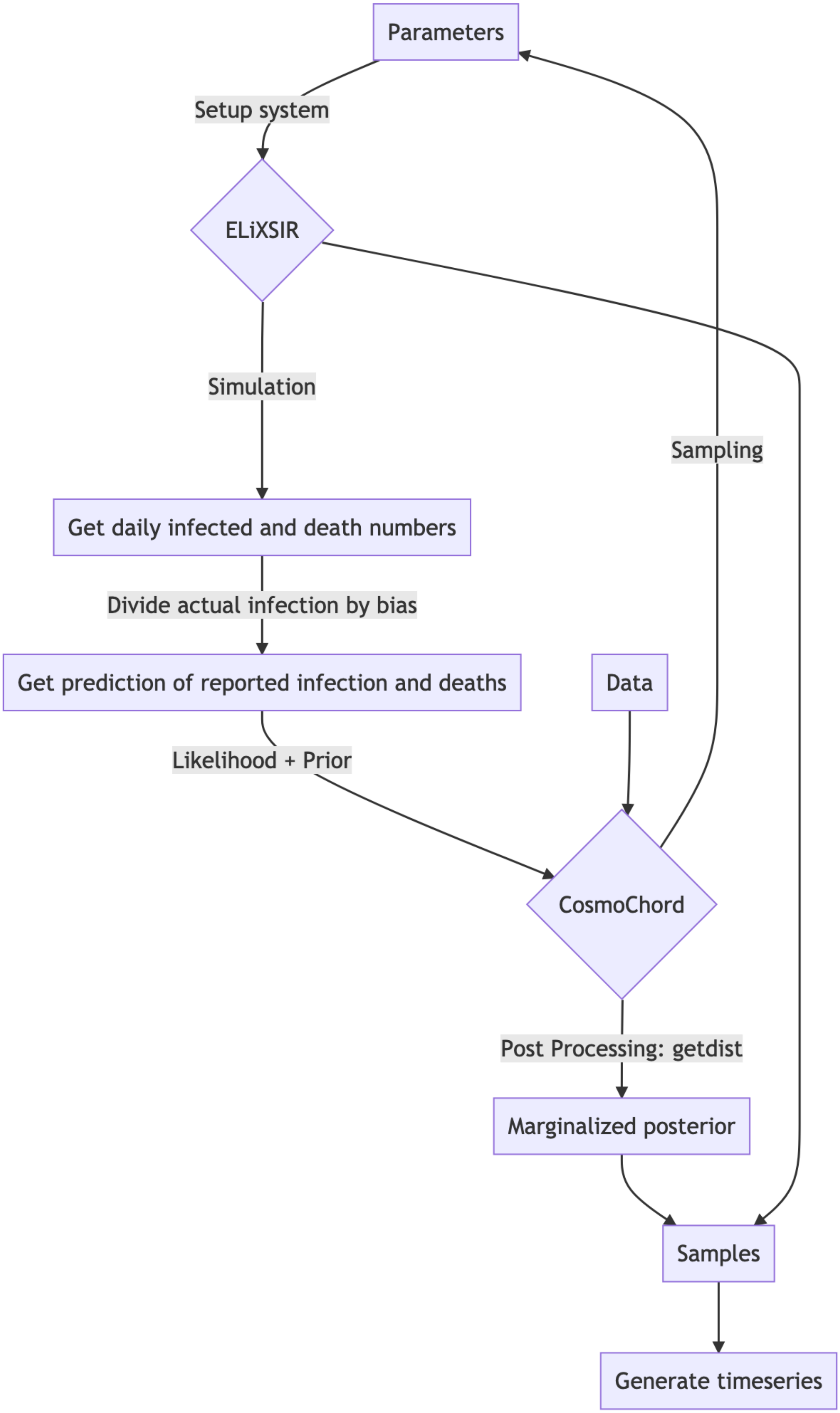

Figure B: Schematic diagram of our analysis.

5 Supporting plots for Karnataka, Mumbai and India (aggregate) analysis

5.1 Posterior distributions and correlations between parameters

Using getdist we compute the posterior distributions of the parameters for different regions and they are presented in triangle plots. We also tabulate the 2σ bounds on the parameters in the following tables.

5.2 Plots for cumulative infection and deaths

The timeseries of daily infection and deaths generate cumulative infection and deaths. In the following plots we plot the bounds on cumulative infection and deaths. Note that since we fit the daily infection and death reports, the error parameters correspond to those values and not the cumulative values.

5.3 Karnataka

Estimations for cumulative infections and deaths are plotted with data in Fig C (without death multiplier) and Fig D (with death multiplier). Marginalized posteriors of the parameters are shown as triangular plot in Fig F while the table showing constraints on parameter values are shown in Fig A.

Parameter	95%	limits	
	Without multiplier	With multiplier 2.2	With multiplier 5
$E_{initial}$	$[557, 957]$	$[739, 1139]$	$[1697 - 2584]$
β_1	$[0.0521, 0.055]$	$[0.05135, 0.05282]$	$[0.050 - 0.052]$
β_2	$[0.0375, 0.0433]$	$[0.044, 0.0469]$	$[0.044 - 0.047]$
β_3	$[0.0433, 0.0545]$	$[0.0556, 0.064]$	$[0.054 - 0.061]$
β_4	—	—	—
τ_1	> 501	> 631	> 646
$Node_1$	$[118.7, 128.5]$	$[106, 118.6]$	$[100 - 114]$
$Node_2$	$[251, 278]$	$[242.9, 258.6]$	$[235 - 257]$
$Node_3$	> 322	> 321	> 321
Δ_{IFR}	$[62, 102]$	< 46.4	$[150 - 282]$
D_{IFR}	< 92.2	$[156.2, 172.5]$	> 287
b	$[83, 123]$	$[99, 119]$	> 172
Δ_b	$[124, 164]$	$[191, 251]$	$[106 - 123]$
σ_1	$[0.365, 0.429]$	$[0.353, 0.409]$	$[0.037 - 0.043]$
σ_2	$[0.464, 0.547]$	$[0.452, 0.533]$	$[0.052 - 0.062]$

Table A: Karnataka: Constraints on parameters. Corresponding to Fig F the 95% constraints and bounds are provided. Two columns on constraints represent the results when we use reported death data without and with undercounting multiplier 2.2 and 5 respectively. Please refer to Table 3 in the main text and Section 1.8 and 1.9 in the main text for detailed descriptions of the symbols.

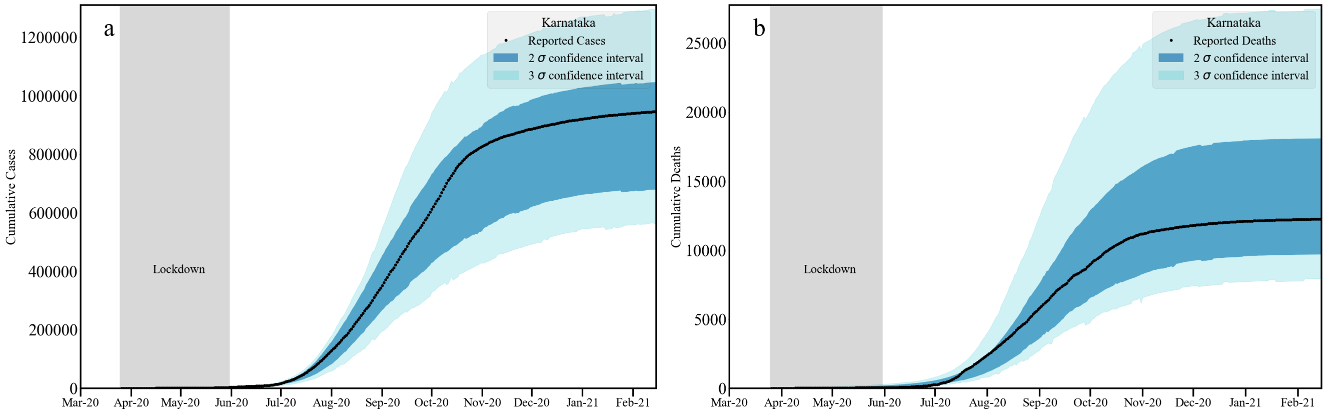

Figure C: Karnataka: Bounds on cumulative infection [a: left] and deaths [b: right] from our analysis plotted with reported data.

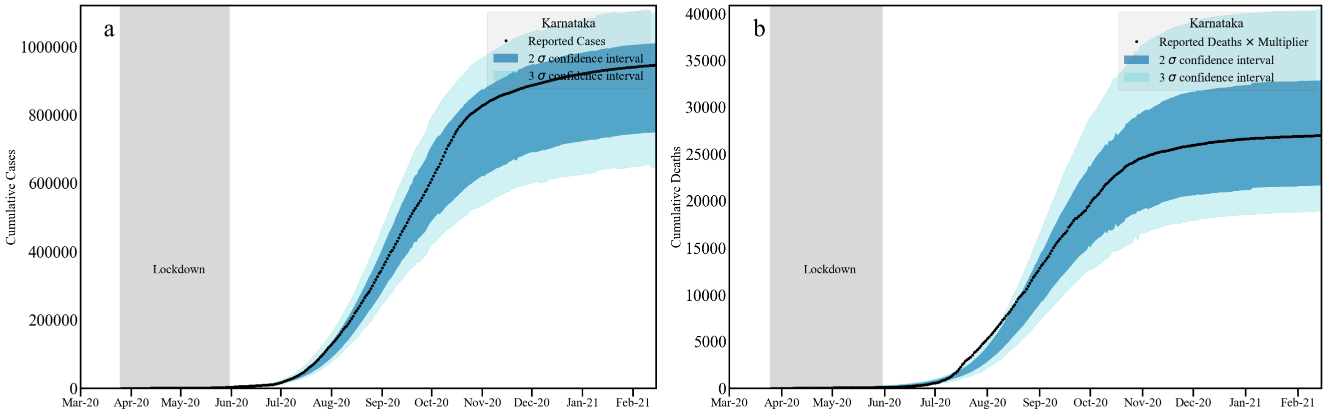

Figure D: Karnataka: Bounds on cumulative infection [a: left] and deaths [b: right] from our analysis plotted with reported data. Note that here death multiplier 2.2 is used to take into account possible death undercounting.

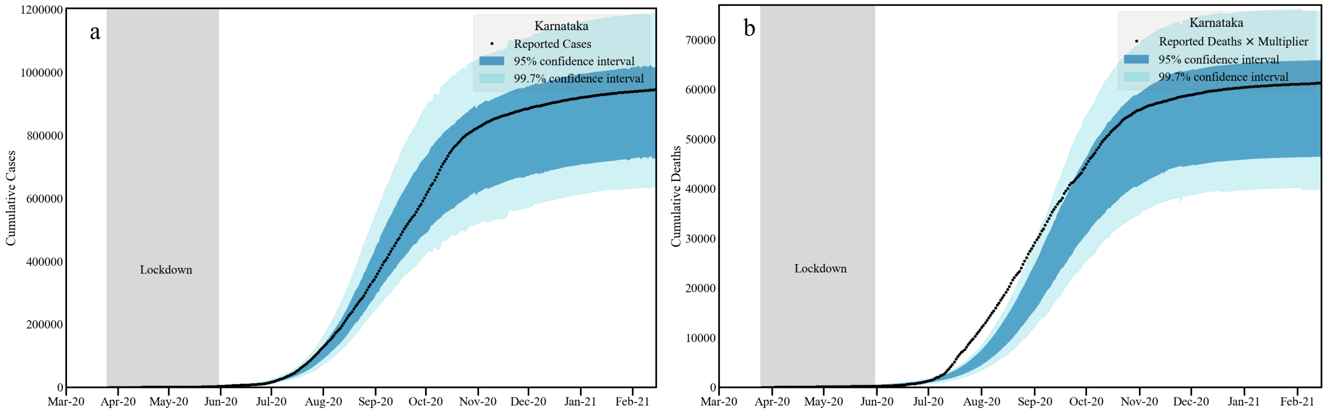

Figure E: Karnataka: Bounds on cumulative infection [a: left] and deaths [b: right] from our analysis plotted with reported data. Note that here death multiplier 5 is used to take into account possible death undercounting.

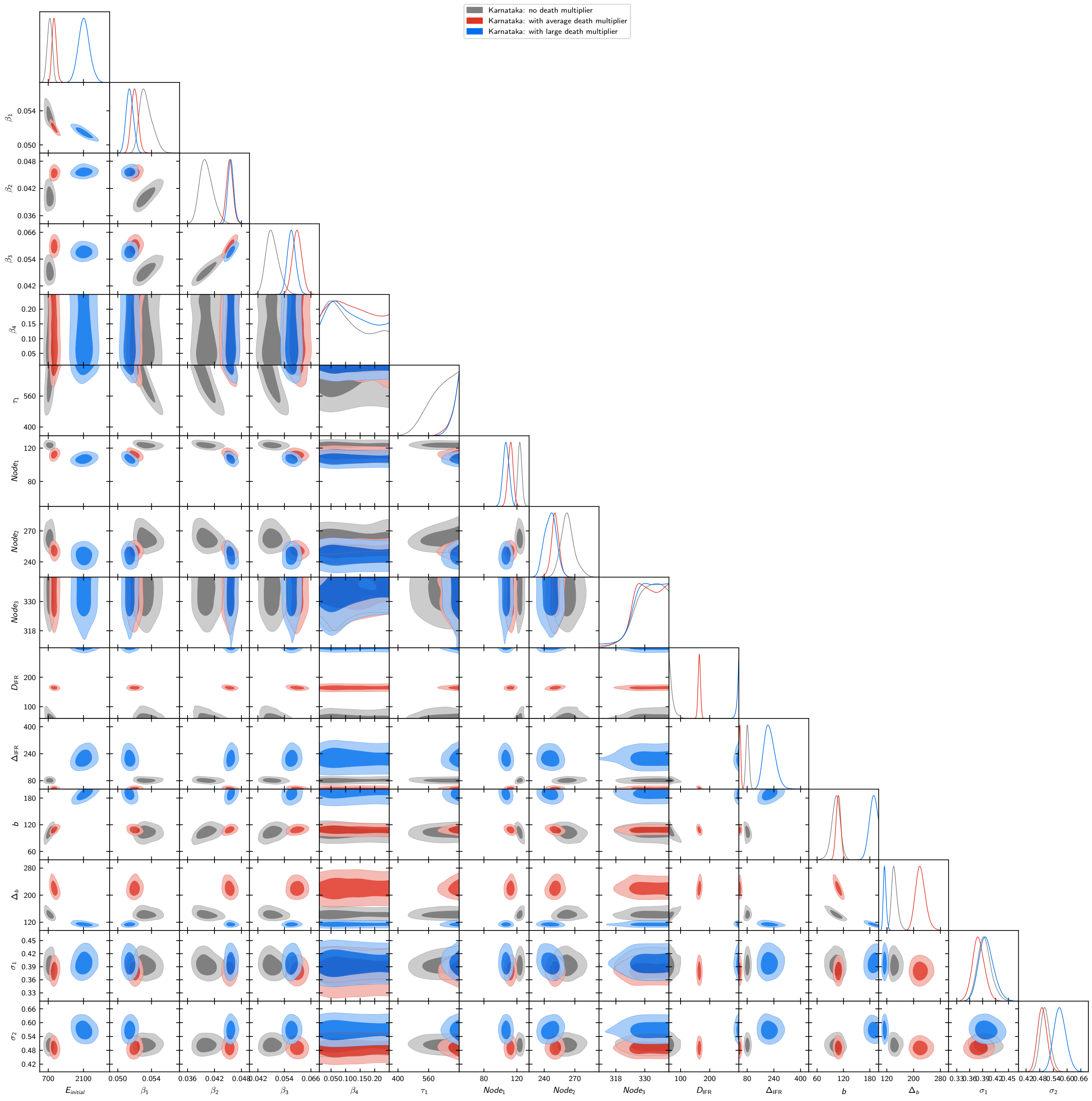

Figure F: Karnataka: Marginalized posteriors of the parameters in the adaptive parametrization in the INDSCI-SIM model against the Karnataka data. We have plotted the constraints obtained assuming and without assuming death multiplier for undercounting. We use death multipliers of 1, 2.2 and 5 to consider the following scenarios – no death undercounting, average and high undercounting obtained from districts. Please refer to Table 3 in the main text and Section 1.8 and 1.9 in the main text for detailed descriptions of the symbols.

5.4 Mumbai

Estimations for cumulative infections and deaths are plotted with data in Fig G. Marginalized posteriors of the parameters are shown as triangular plot in Fig H while the table showing constraints on parameter values are shown in Fig B.

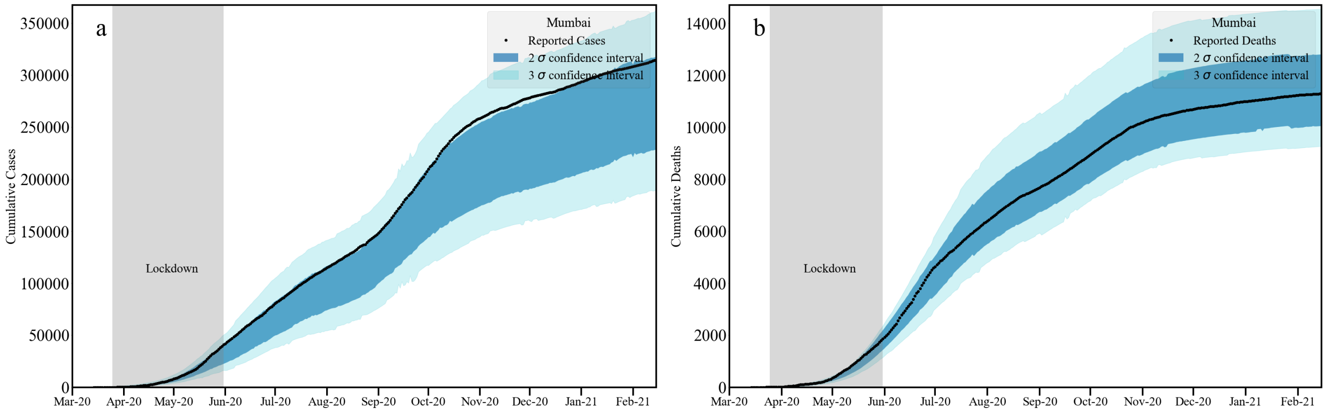

Figure G: Mumbai: Bounds on cumulative infection [a: left] and deaths [b: right] from our analysis plotted with reported data.

Parameter	95% limits
$E_{initial}$	$[474, 874]$
β_1	$[0.0662, 0.0727]$
β_2	$[0.0408, 0.0478]$
β_3	$[0.073, 0.098]$
β_4	$[0.12, 0.183]$
τ_1	> 388
$Node_1$	$[55.2, 66.1]$
$Node_2$	$[150.4, 156.4]$
$Node_3$	< 254
Δ_{IFR}	$[43, 63]$
D_{IFR}	< 94.3
b	$[142, 171]$
Δ_b	$[188, 228]$
σ_1	$[0.433, 0.51]$
σ_2	$[0.31, 0.372]$

Table B: Mumbai: Constraints on parameters. Corresponding to Fig H the 95% constraints and bounds are provided. Please refer to Table 3 in the main text and Section 1.8 and 1.9 in the main text for detailed descriptions of the symbols.

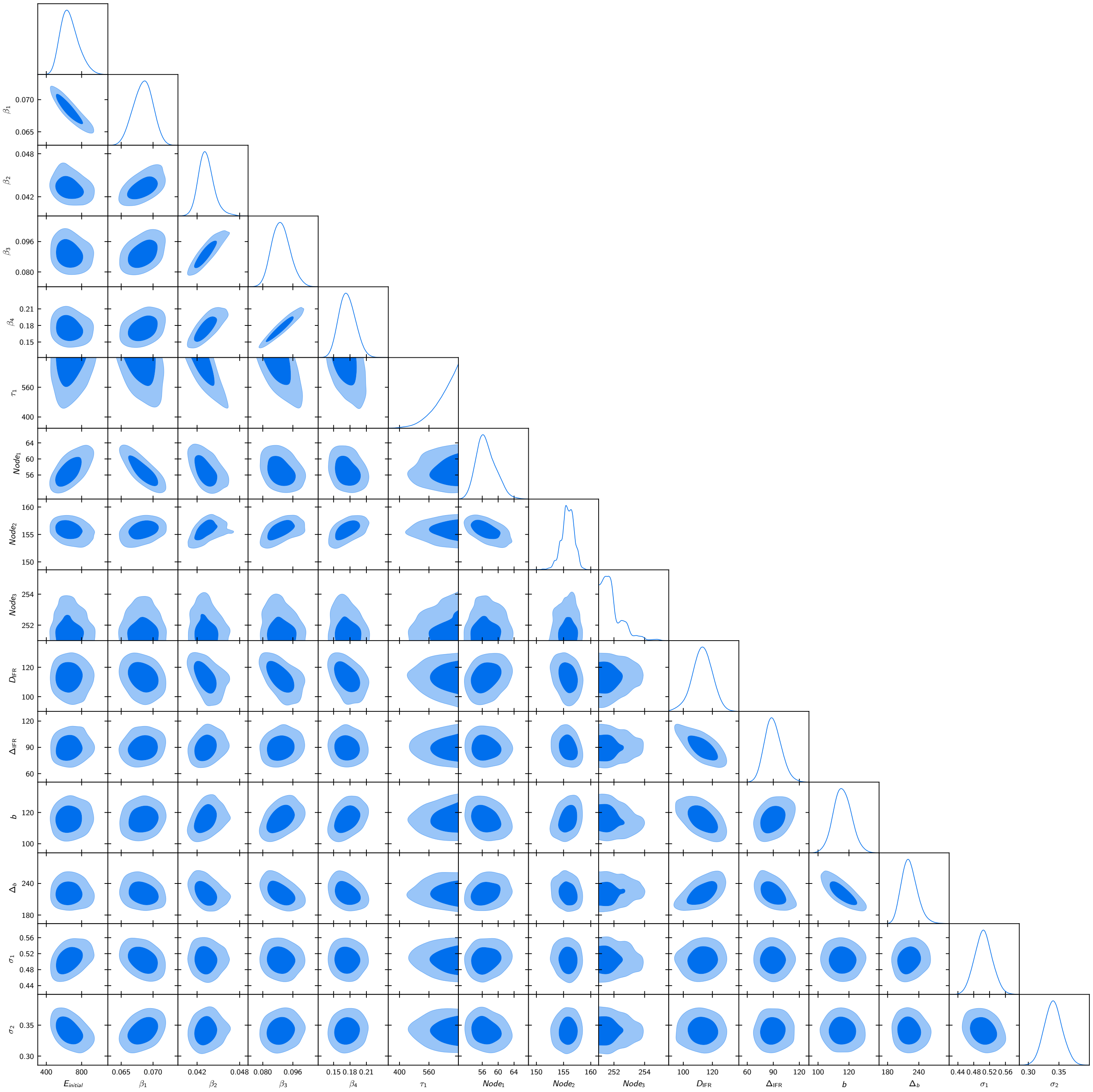

Figure H: Mumbai: Marginalized posteriors of the parameters in the adaptive parametrization in the INDSCI-SIM model against the Mumbai data. Please refer to Table 3 in the main text and Section 1.8 and 1.9 in the main text for detailed descriptions of the symbols.

Parameter	95% limits
$E_{initial}$	[7580, 5680]
β_1	[0.0734, 0.0775]
β_2	[0.1097, 0.1241]
β_3	[0.201, 0.239]
τ_1	[133.4, 151.8]
$Node_1$	[149.7, 158.1]
$Node_2$	[230.6, 237.3]
Δ_{IFR}	[52, 72]
D_{IFR}	[101.4, 115.9]
b	> 194
Δ_b	[230, 261]
σ_1	[0.337, 0.396]
σ_2	[0.218, 0.258]

Table C: India: Mean and 95% bounds on the parameters. These values correspond to the posteriors plotted in Fig J. Please refer to Table 3 in the main text and Section 1.8 and 1.9 in the main text for detailed descriptions of the symbols.

5.5 India

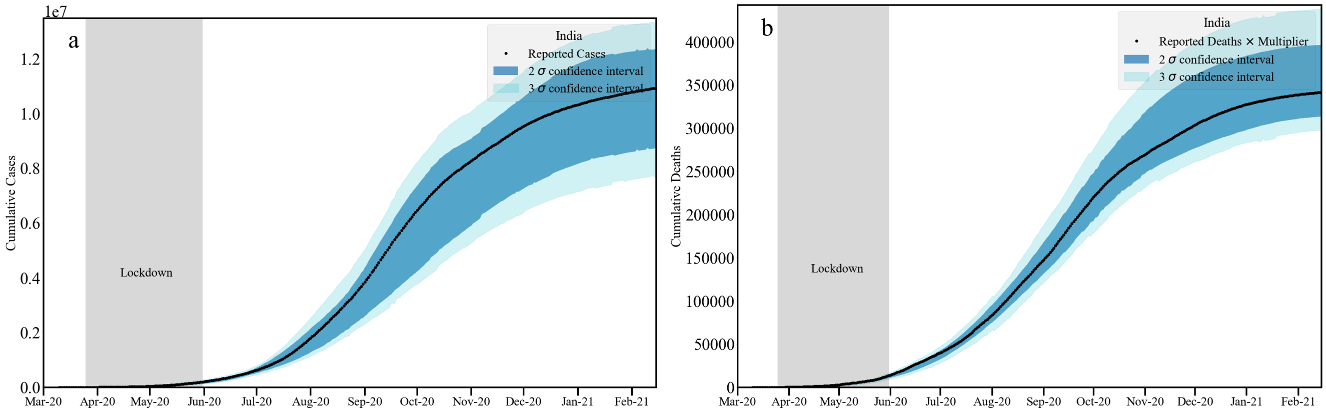

Figure I: India: Bounds on cumulative infection [a: left] and deaths [b: right] from our analysis plotted with reported data. Note that here death multiplier 2.2 is used to take into account possible death undercounting.

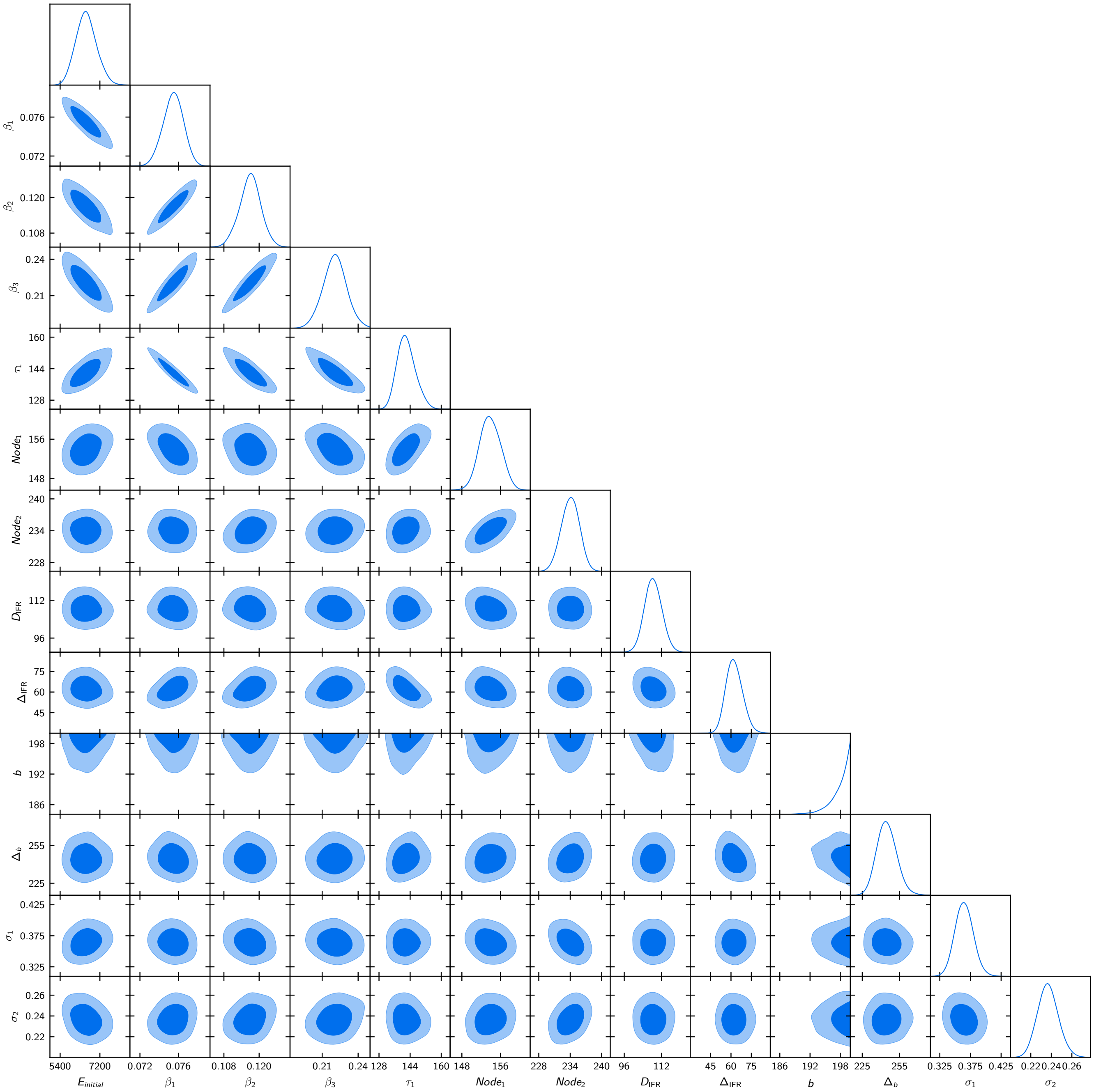

Figure J: India: Marginalized posteriors of the parameters in the adaptive parametrization for India-level data. Please refer to Table 3 in the main text and Section 1.8 and 1.9 in the main text for detailed descriptions of the symbols.

6 Infection curves for select Indian cities

In the main text, we presented the analysis of Mumbai alone. Here, following the same protocol, we describe results for Bengaluru Urban, Chennai, Pune, and Delhi.

6.1 Bengaluru Urban

Our analysis for Bengaluru Urban is presented in Fig K; posterior distributions and parameter constraints are provided in SI: Section 3, Fig 2 and Table 1). For the calculations, the population data for Bangalore is sourced from the projections for the period 2020-2021, as provided by the report issued by Directorate of Economics and Statistics, Bangalore [9]. Our approach yields a double-peak structure in the curve of daily infections, reflecting a temporary flattening of the curve around September 2020 followed by an increase that resulted in a peak around October. Between mid-September and mid-October, we observe a plateau in deaths. Our results can be compared to those from serosurveys, as quoted in Refs. [10, 11, 12]. In September of 2020, these calculations yield a seroprevalence in Bengaluru Urban of about 30%, consistent with serosurvey results.

We note that the model indicates that a substantial fraction of the population in Bengaluru had already been infected by Feb 15. The effective reproductive ratio $R(t)$ decreases abruptly during the lockdown, while displaying an equally sharp increase post lockdown. Our estimate for those infected in Bengaluru city by Feb 15, 2021 lies between 64% and 85% at the 95% confidence level. The bias multiplier narrows quite rapidly, being approximately 10 by mid-February 2021; at the time of the Bengaluru serosurvey, this factor was approximately 30. Our estimate for the IFR at the end of the first wave indicates a value in the vicinity of 0.05%, roughly consistent with similar estimates for the first wave based on serosurveys. An increase in the IFR, say to 0.08 or 0.1, can also be achieved if we assume that deaths have been under-counted by a suitable factor, while leaving our estimates for total infections the same.

Estimations for cumulative infections and deaths are plotted with data in Fig L. Marginalized posteriors of the parameters are shown as triangular plot in Fig M while the table showing constraints on parameter values are shown in Fig D.

Parameter	95% limits
$E_{initial}$	$[4805, 15805]$
β_1	$[0.0104, 0.0233]$
β_2	$[0.0675, 0.0745]$
β_3	$[0.1196, 0.1384]$
β_4	> 0.218
τ_1	$[153, 203]$
$Node_1$	$[11.7, 17.4]$
$Node_2$	$[138.6, 145]$
$Node_3$	$[208, 220.9]$
Δ_{IFR}	< 33.1
D_{IFR}	$[87.8, 103.3]$
b	$[78, 98]$
Δ_b	$[107, 147]$
σ_1	$[0.471, 0.556]$
σ_2	$[0.537, 0.637]$

Table D: Bengaluru Urban: Constraints on parameters. Corresponding to Fig M the 95% constraints and bounds are provided. Please refer to Table 3 in the main text and Section 1.8 and 1.9 in the main text for detailed descriptions of the symbols.

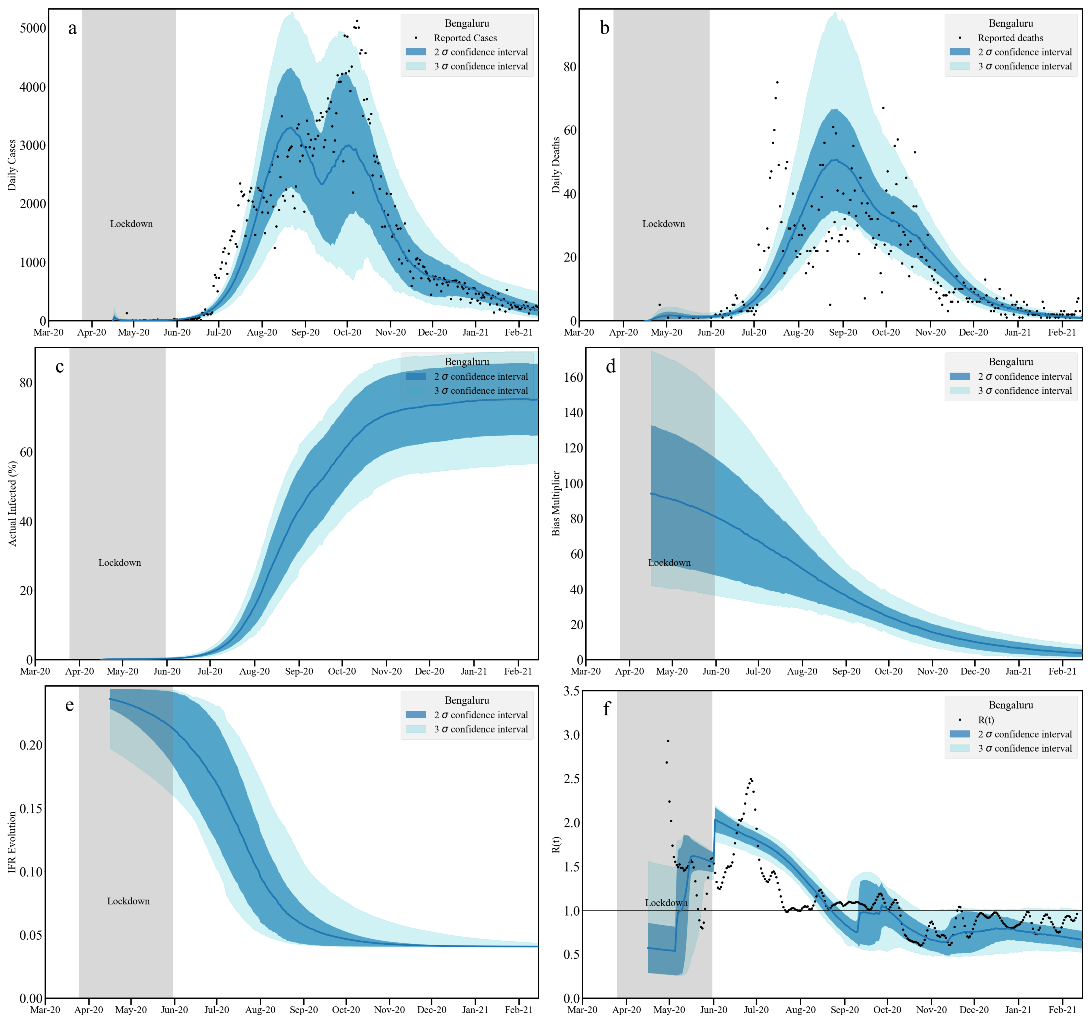

Figure K: Bengaluru Urban: Timeseries analysis for Bengaluru Urban. We plot the fit to the daily infected cases [a: top left] and daily reported deaths [b: top right] assuming no death undercounting. The middle panel contains the cumulative actual infected cases [c: left] and the bias multiplicative factor [d: right] obtained as a ratio between actual and reported infections. The left plot [e] at the bottom panel contains the evolution of the age averaged IFR. The bottom right plot [f] contains our estimation of $R(t)$ and an independent [1] measurement. Note that the bands correspond to 2σ and 3σ confidence levels.

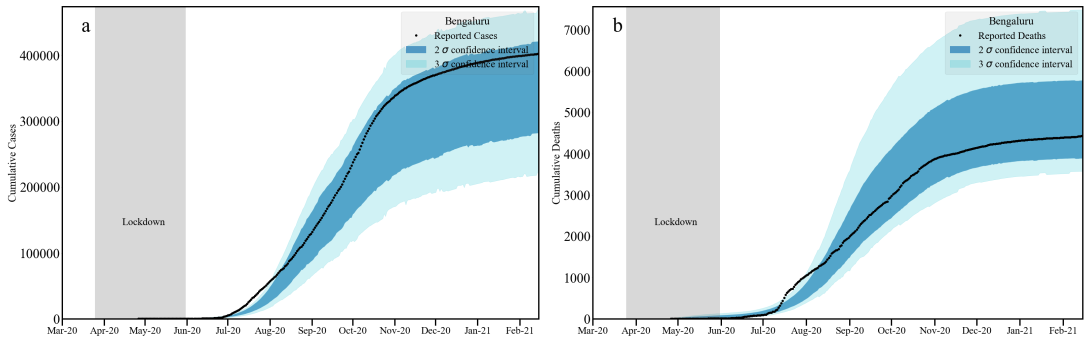

Figure L: Bengaluru Urban: Bounds on cumulative infection [a: left] and deaths [b: right] from our analysis plotted with reported data.

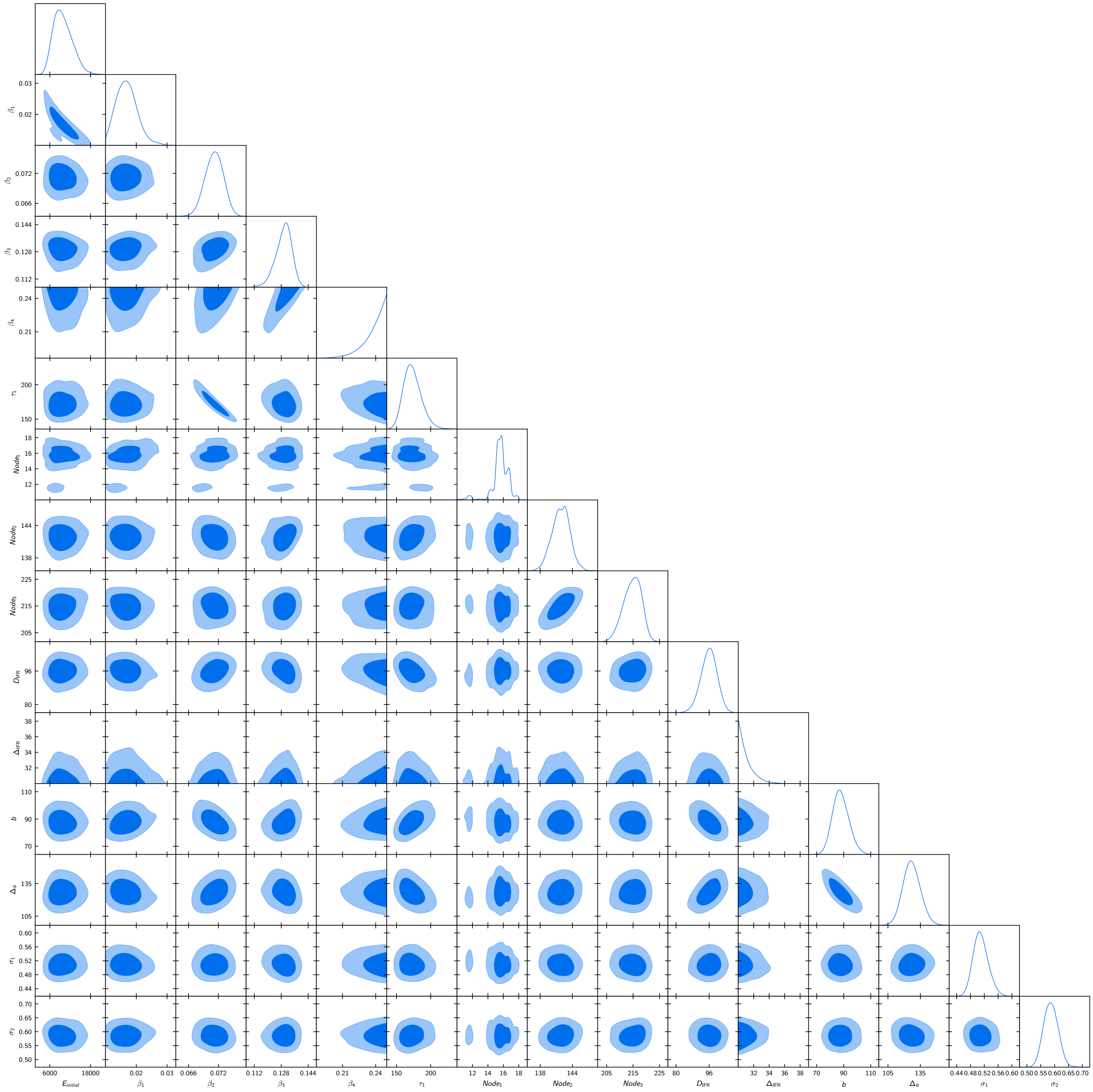

Figure M: Bengaluru Urban: Marginalized posteriors of the parameters in the adaptive parametrization in the INDSCI-SIM model against the Bengaluru Urban data. Please refer to Table 3 in the main text and Section 1.8 and 1.9 in the main text for detailed descriptions of the symbols.

6.2 Chennai

Our results for Chennai are plotted in Fig N, with posterior distributions and parameter constraints provided in the SI: Section 3, Fig 3 and Table 2). For these calculations, we source population data for Chennai from Wikipedia, which takes into account the expansion of the district limits in 2018 [13]. Apart from the national lockdown of 68 days, we note that Chennai had imposed a shorter, local lockdown during the period June 19 - July 5, 2020. Both lockdown periods are marked in grey. While across the first lockdown the numbers of infected increased steadily, the numbers began to show a decline following the second lockdown. We see minor peaks around mid-August and October of a lesser height when compared to the first peak. This decrease is also corroborated by the estimates for the $R(t)$. Serosurvey results for Chennai have been reported in Ref. [14].

There are clearly issues with data here since around July 2020, the peak in deaths appears to precede the infection peak, although the data is quite noisy. After that, a steady decrease in the death numbers is seen. While between mid-July to end-October the infection numbers change only marginally, the numbers of deaths reduce largely monotonically. This suggests an effective decrease in IFR, perhaps related to improvements in patient management. The bias multiplier also show a decrease. However the rate of the decline is much slower compared to Bengaluru Urban. The plot indicates a 30-fold undercounting of cases initially. This is reduced to around 15 by the middle of February 2020. A low test positivity since the onset of infection is supported by this plot and attributed to a large scale testing program. The actual infection plot suggests that nearly 54-85% (at 95% C.L.) of the Chennai population has been infected by mid-February.

We can compare these predictions to data from Ref. [15] for the state of Tamil Nadu as a whole in December 2020, which found that seroprevalence in urban areas (36.9%) was higher than in rural areas (26.9%). They found that 22.6 million persons were infected by the end of November, roughly 36 times the number of confirmed cases. For Chennai, the estimated seroprevalance by December was in the vicinity of 40%, within the 95% bound of our own results.

Estimations for cumulative infections and deaths are plotted with data in Fig O. Marginalized posteriors of the parameters are shown as triangular plot in Fig P while the table showing constraints on parameter values are shown in Fig E.

Parameter	95% limits
$E_{initial}$	$[128, 228]$
β_1	$[0.0664, 0.0711]$
β_2	$[0.0404, 0.0456]$
β_3	$[0.0596, 0.0759]$
β_4	$[0.076, 0.107]$
β_5	< 0.158
τ_1	> 516
$Node_1$	$[63.3, 70.5]$
$Node_2$	$[142.4, 155.2]$
$Node_3$	$[220.1, 237.5]$
$Node_4$	$[282, 334]$
Δ_{IFR}	$[73, 123]$
D_{IFR}	$[65, 105]$
b	$[40, 50]$
Δ_b	> 379
σ_1	$[0.321, 0.38]$
σ_2	$[0.437, 0.514]$

Table E: Chennai: Constraints on parameters. Corresponding to Fig P the 95% constraints and bounds are provided. Please refer to Table 3 in the main text and Section 1.8 and 1.9 in the main text for detailed descriptions of the symbols.

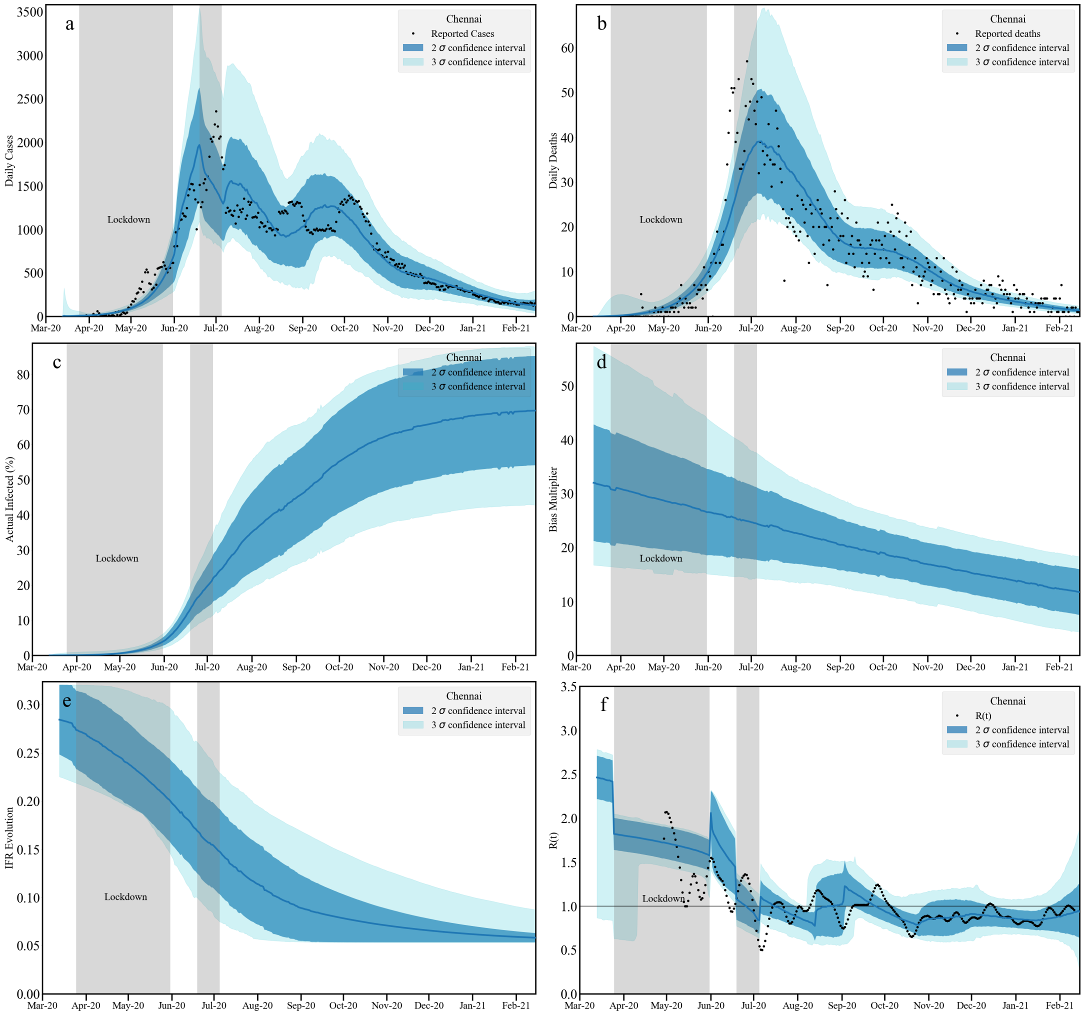

Figure N: Chennai: Timeseries analysis for Chennai. We plot the fit to the daily infected cases [a: top left] and daily reported deaths [b: top right] assuming no death undercounting. Middle panel contains the cumulative actual infected cases [c: left] and the bias multiplicative factor [d: right] obtained as a ratio between actual and reported infections. The left plot [e] at the bottom panel contains the evolution of age averaged IFR. The bottom right plot [f] contains our estimation of $R(t)$ and an independent [1] measurement. Note that the bands correspond to 2σ and 3σ confidence levels.

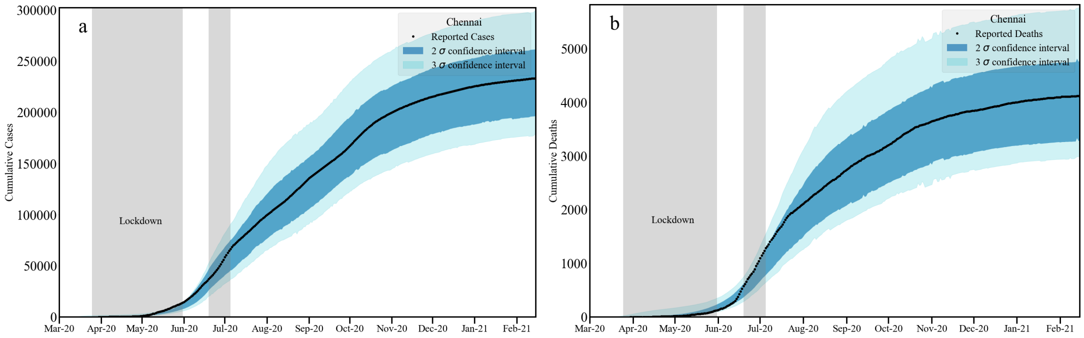

Figure O: Chennai: Bounds on cumulative infection [a: left] and deaths [b: right] from our analysis plotted with reported data.

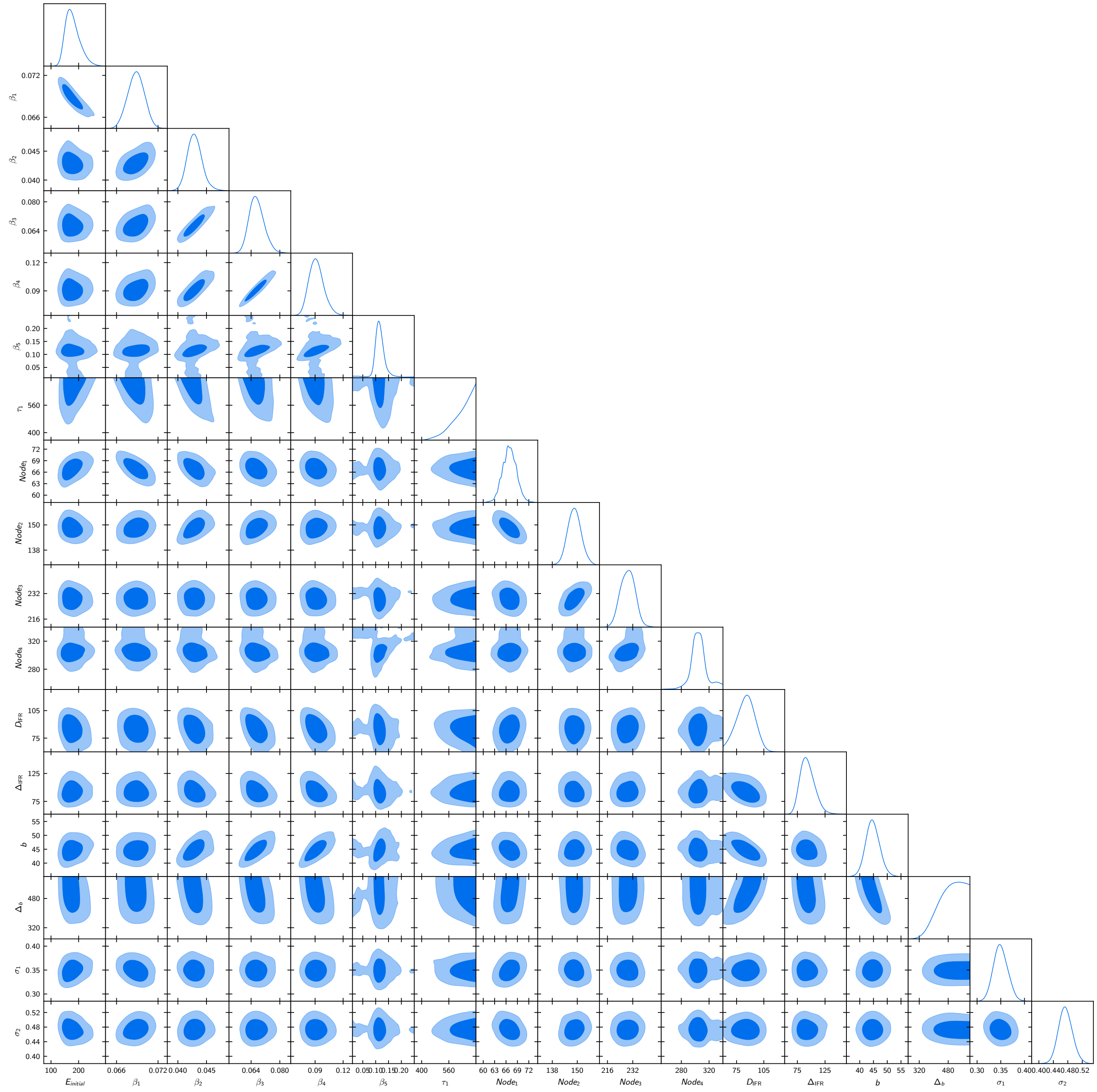

Figure P: Chennai: Marginalized posteriors of the parameters in the adaptive parametrization in the INDSCI-SIM model against the Chennai data. Please refer to Table 3 in the main text and Section 1.8 and 1.9 in the main text for detailed descriptions of the symbols.

6.3 Delhi

Fig Q present our analysis with the Delhi data. The data for Delhi presents a challenge to modelling because of the presence of multiple peaks. The data requires 5 adaptive windows to fit. Within these 5 windows, the constrained infectivity parameter β show a oscillatory trend. This oscillatory pattern may possibly reflect patterns in the return of migrant workers to the national capital region. A number of different serosurvey results have been reported for Delhi, including in Ref. [16]. For our calculations, we use Delhi's population data from the projections for the year 2020 reported in the 2019 report published by the National Commission on Population [17].

With these windows, our model captures the trends in the data. Posterior distributions and parameter constraints are provided in the SI: Section 3, Fig 4 and Table 3. We find multi-peak posteriors in the *Node* parameters reflecting several possible solutions of the Delhi trajectory. The reported deaths here show outliers around June 2020 that occur before infection peaks towards the middle and end of June. Although the IFR decreases we find that it is still consistent with 0.1% IFR around February 2021. The bias multiplier settles down around 20, indicating that a point estimate of about 80% (71-90% at 95% C.L.) infected population in Delhi by January 2021, with a confidence of interval of ±10%. Our limits at the 3σ confidence limit though indicate a range of 50-90% infected, given the skewed posterior distribution of bias. The estimated $R(t)$ as expected show an oscillatory behavior, consistent with the independent estimates. We observe that $R(t)$ fluctuates in a range $R(t) \sim 1 - 1.5$ before it reduces below 1 around December 2020.

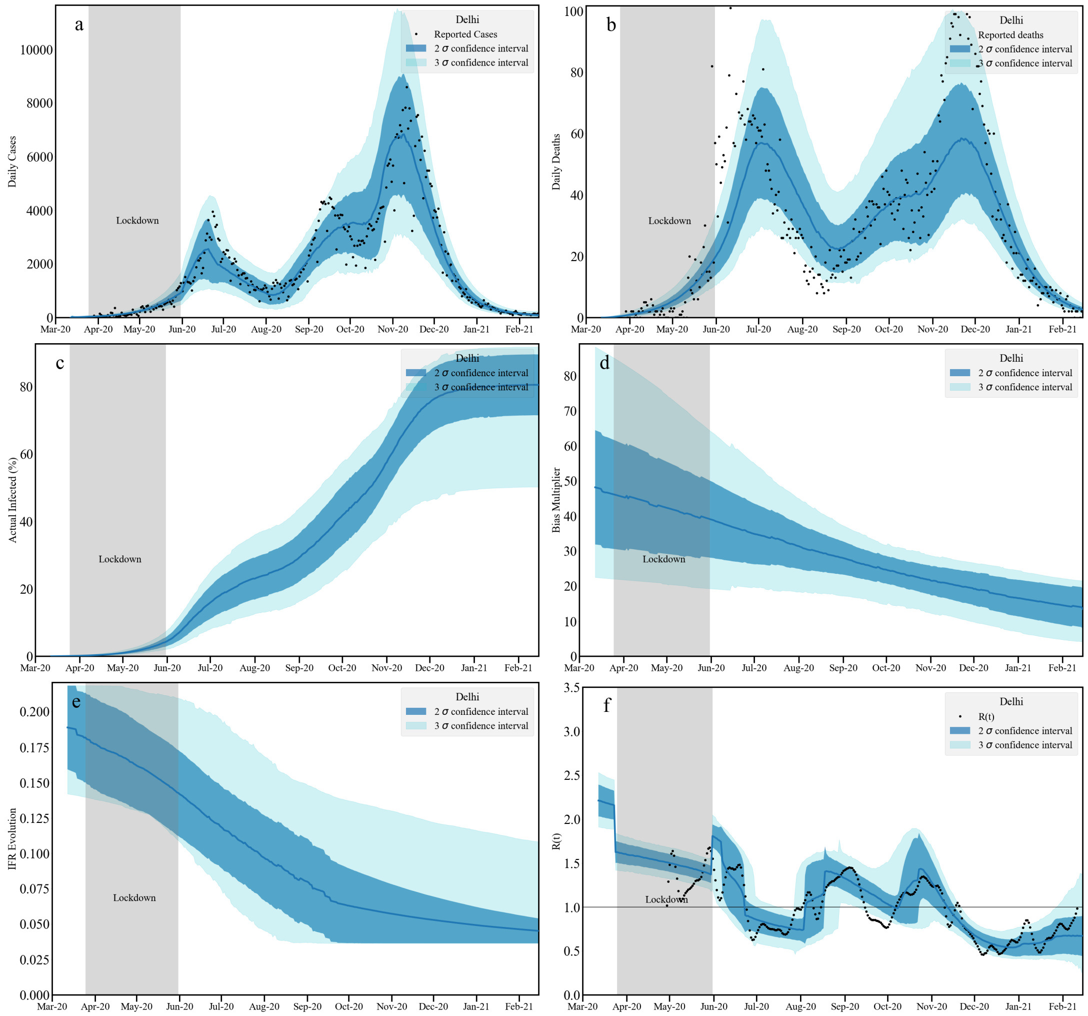

Figure Q: Delhi: Timeseries analysis for Delhi. We plot the fit to the daily infected cases [a: top left] and daily reported deaths [b: top right] assuming no death undercounting. Middle panel contains the cumulative actual infected cases [c: left] and the bias multiplicative factor [d: right] obtained as a ratio between actual and reported infections. The left plot [e] at the bottom panel contains the evolution of age averaged IFR. The bottom right plot [f] contains our estimation of $R(t)$ and an independent [1] measurement. Note that the bands correspond to 2σ and 3σ confidence levels.

Results from the fifth serosurvey in Delhi concluded that about 56% of the over 28,000 people whose blood samples were

collected in January 2021 had developed antibodies against COVID-19. The first such survey done in the city in June-July had shown that 23.4% of people surveyed had developed antibodies against the virus. Similar surveys in August showed that 29.1% of people had antibodies to SARS-CoV-2 at that time. This became 25.1% in September and 25.5% in October. These results are approximately consistent with the results we present here, except for the January serosurvey, which lies outside our 95% confidence interval. However, due to the decay of antibodies, seroprevalence as derived from CLIA tests are expected to underestimate numbers of infected. We expect that accounting for this decay would yield closer agreement. Initial estimates of Delhi's IFR in Ref. [18] yield numbers consistent with a range (0.05-0.1%), consistent with our calculation here. However, other calculations yield a cumulative proportion of the population estimated infected of 48.7% (95% CrI 22.1% – 76.8%) by end-September 2020. These are noticeably larger than our own estimates of about 30% at that time. (The IFR assumed in that work was considerably larger, though, between 0.22% and 0.39%; this required that a relatively small number of deaths were actually recorded, about 28% of the actual number if the IFR was 0.21%.).

Estimations for cumulative infections and deaths are plotted with data in Fig R. Marginalized posteriors of the parameters are shown as triangular plot in Fig S while the table showing constraints on parameter values are shown in Fig F.

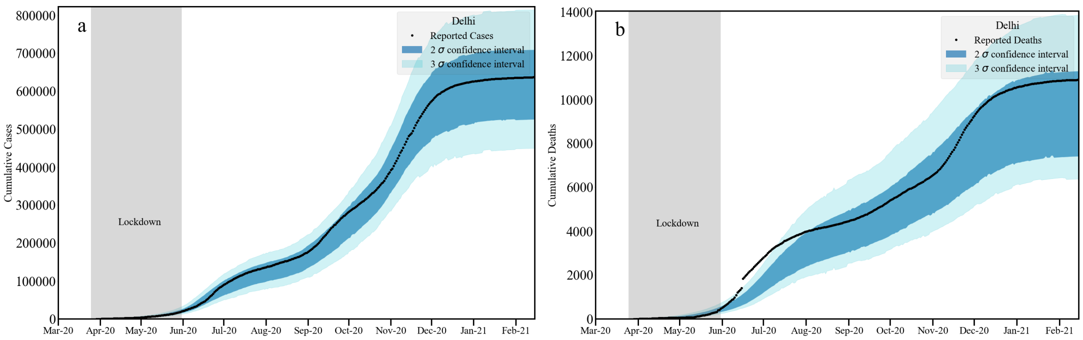

Figure R: Delhi: Bounds on cumulative infection [a: left] and deaths [b: right] from our analysis plotted with reported data.

Parameter	95% limits
$E_{initial}$	$[2299, 4199]$
β_1	$[0.0553, 0.0595]$
β_2	$[0.0291, 0.0345]$
β_3	$[0.0596, 0.0719]$
β_4	$[0.111, 0.149]$
β_5	$[0.174, 0.248]$
τ_1	> 439
$Node_1$	$[74.3, 83]$
$Node_2$	$[131.2, 138.3]$
$Node_3$	$[202.6, 207]$
$Node_4$	$[278, 306]$
Δ_{IFR}	$[90, 150]$
D_{IFR}	< 102
b	$[65, 82]$
Δ_b	$[218, 378]$
σ_1	$[0.302, 0.359]$
σ_2	$[0.479, 0.57]$

Table F: Delhi: Constraints on parameters. Corresponding to Fig S the 95% constraints and bounds are provided. Please refer to Table 3 in the main text and Section 1.8 and 1.9 in the main text for detailed descriptions of the symbols.

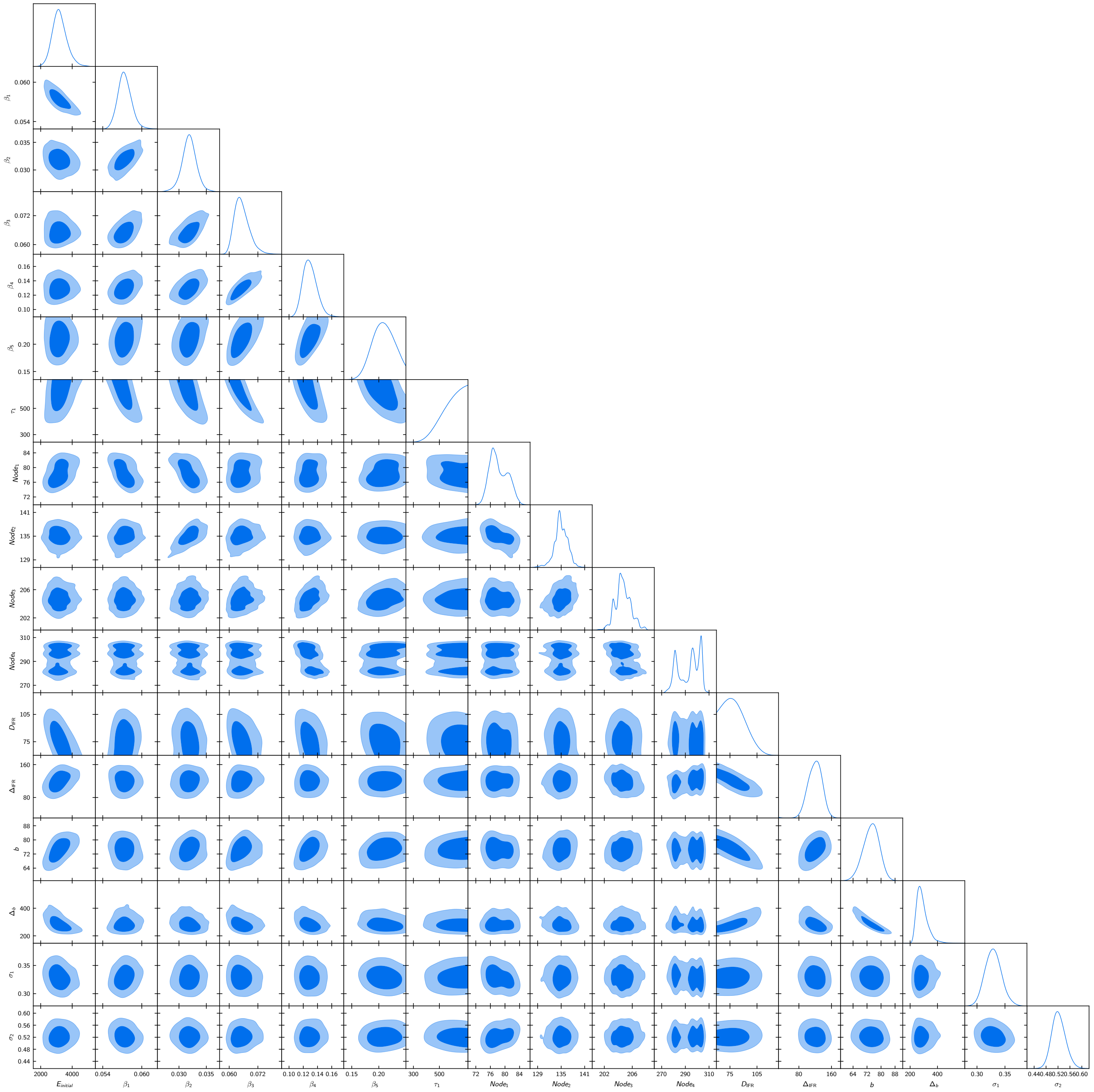

Figure S: Delhi: Marginalized posteriors of the parameters in the adaptive parametrization in the INDSCI-SIM model against the Delhi data. Please refer to Table 3 in the main text and Section 1.8 and 1.9 in the main text for detailed descriptions of the symbols.

6.4 Pune

In this section we present our final city analysis, that of Pune in Fig T. The population of the district is taken as per projection for 2021 [19]. The posterior distributions and parameter constraints for this calculation are provided in SI: Section 3, Fig 6 and Table 5. As was the case with Chennai, Pune had imposed a local lockdown during 14-24 July 2020. There is substantial scatter in the reported death data, compared to the data for numbers of infected. This may arise from incomplete reporting initially. The infection data show a single prominent peak structure with a possible kink around mid-July 2020. The global peak comes around mid-September 2020, with infected numbers decreasing after that and becoming nearly constant around November. The death reports follow the infection in standard manner. However we do not find a substantial decrease in reported deaths *vis a vis.* infections. This is also reflected in the IFR plot where we see relatively minor changes from the initial IFR. The bias multiplier changes from 35 to 15 indicating an improvement in testing. We also find a large infected fraction, of 64-90% of the population in Pune. Our estimated $R(t)$ stays around 1.5 till September 2020 after which it comes down sharply to nearly 0.5 in two months. In December we see a rise in $R(t)$ which remains nearly constant till the end of the first wave around mid-February. $R(t)$ from December 2020 to February 2021 stays at nearly 1 which can also be observed in the flatness in reported cases and deaths.

A serosurvey in Pune, conducted between 20th July and 5th August 2020, found a seroprevalance of 51·3% (95%CI 39·9 to 62·4) [20] . The overall IFR was calculated to be 0.21. The inferred seroprevalence lies well above our own median prediction of about 35% by August 2020, although our IFR estimates at that time are closer to the value inferred from the serosurveys, at about 0.17. We note, however, that the substantial spread in the inferred IFRs across different localities even within the city make comparisons more difficult. Compartmental models are insensitive to such ultra-local variations; other, more refined individual-based models will be required to assess these effects.

Estimations for cumulative infections and deaths are plotted with data in Fig U. Marginalized posteriors of the parameters are shown as triangular plot in Fig V while the table showing constraints on parameter values are shown in Fig G.

Parameter	95% limits
$E_{initial}$	$[5670, 7670]$
β_1	$[0.0465, 0.049]$
β_2	$[0.0666, 0.0815]$
β_3	$[0.137, 0.2]$
β_4	$[0.164, 0.272]$
τ_1	> 540
$Node_1$	> 119
$Node_2$	$[190.9, 198.7]$
$Node_3$	$[240, 269]$
Δ_{IFR}	< 55.9
D_{IFR}	$[126, 150]$
b	$[45.8, 54.3]$
Δ_b	$[329, 629]$
σ_1	$[0.363, 0.427]$
σ_2	$[0.592, 0.698]$

Table G: Pune: Constraints on parameters. Corresponding to Fig V the 95% constraints and bounds are provided. Please refer to Table 3 in the main text and Section 1.8 and 1.9 in the main text for detailed descriptions of the symbols.

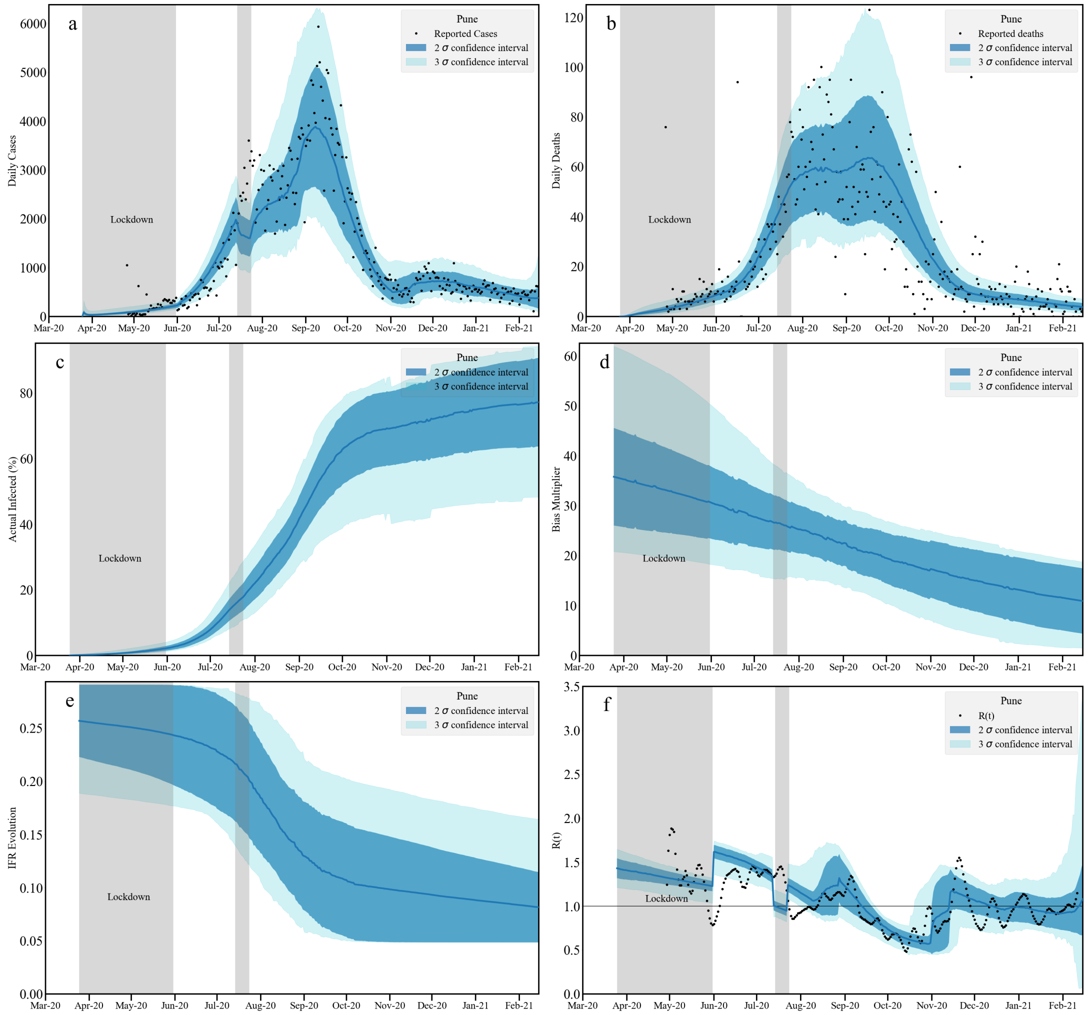

Figure T: Pune: Timeseries analysis for Pune. We plot the fit to the daily infected cases [a: top left] and daily reported deaths [b: top right] assuming no death undercounting. Middle panel contains the cumulative actual infected cases [c: left] and the bias multiplicative factor [d: right] obtained as a ratio between actual and reported infections. The left plot [e] at the bottom panel contains the evolution of age averaged IFR. The bottom right plot [f] contains our estimation of $R(t)$ and an independent [1] measurement. Note that the bands correspond to 2σ and 3σ confidence levels.

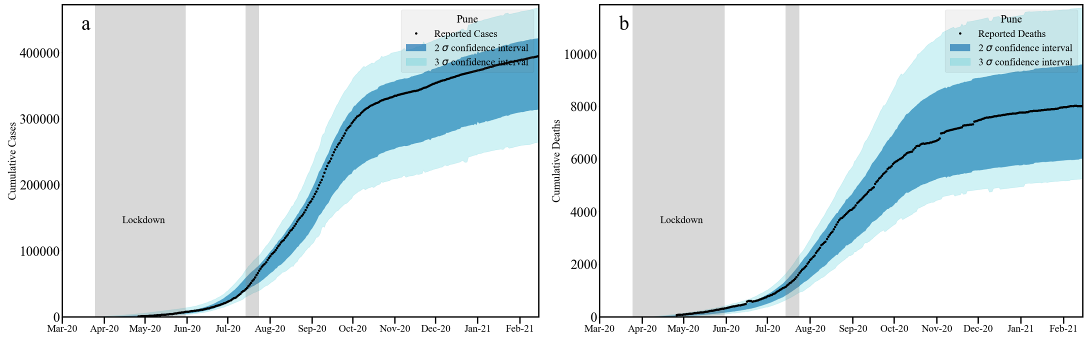

Figure U: Pune: Bounds on cumulative infection [a: left] and deaths [b: right] from our analysis plotted with reported data.

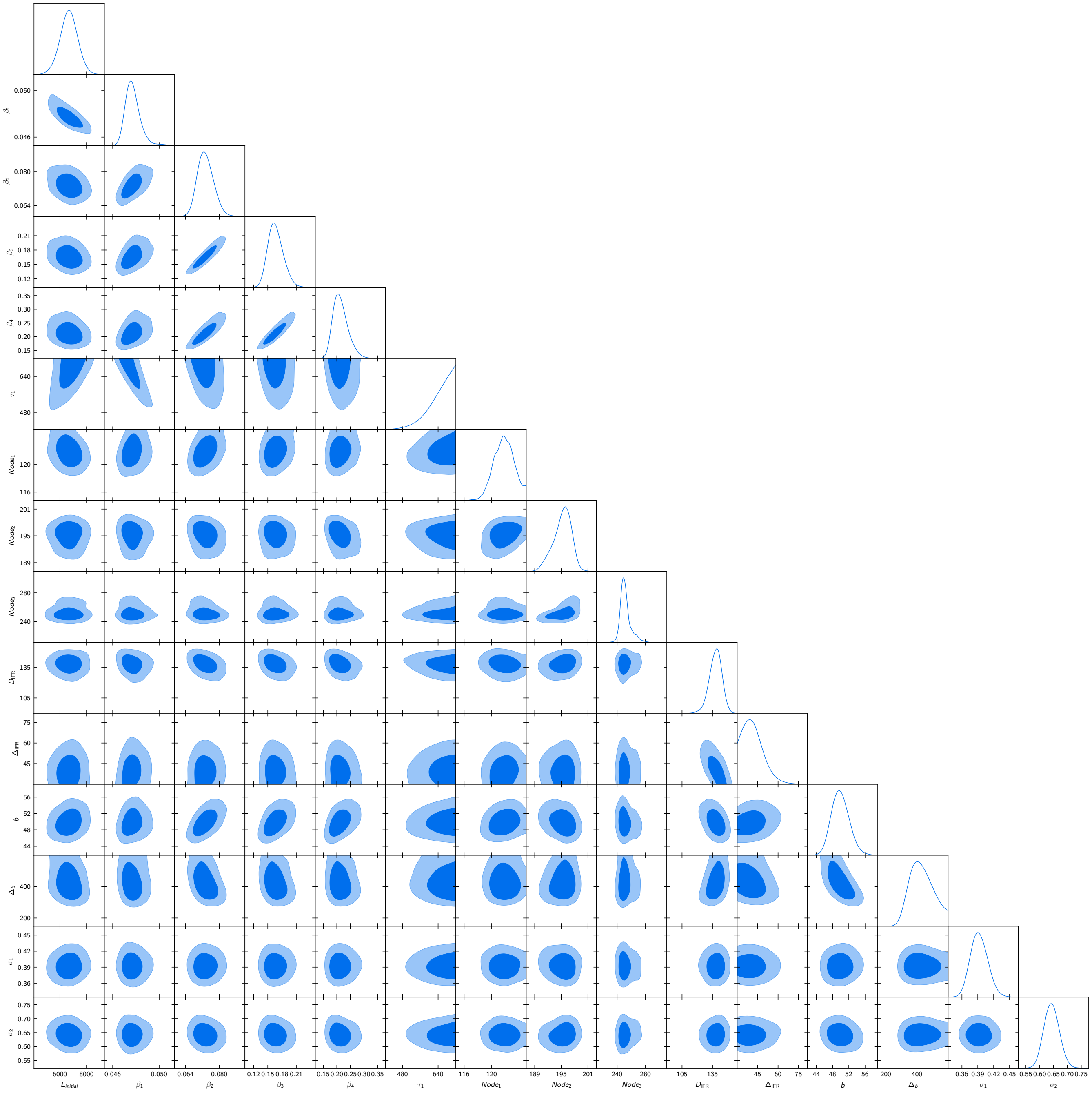

Figure V: Pune: Marginalized posteriors of the parameters in the adaptive parametrization in the INDSCI-SIM model against the Pune data. Please refer to Table 3 in the main text and Section 1.8 and 1.9 in the main text for detailed descriptions of the symbols.

References

[1] Gruisen G, Lerer A. k-sys/covid-19; 2021. Available from: https://github.com/k-sys/covid-19/blob/fac27a226d907bf59a9205a59660d2f281621ad9/Realtime%20R0.ipynb.

[2] Keeling MJ, Rohani P. Modeling infectious diseases in humans and animals. Princeton university press; 2011.

[3] Diekmann O, Heesterbeek JaP, Roberts MG. The construction of next-generation matrices for compartmental epidemic models. Journal of The Royal Society Interface. 2010;7(47):873–885. doi:10.1098/rsif.2009.0386.

[4] Prem K, Cook AR, Jit M. Projecting social contact matrices in 152 countries using contact surveys and demographic data. PLOS Computational Biology. 2017;13(9):1–21. doi:10.1371/journal.pcbi.1005697.

[5] Handley WJ, Hobson MP, Lasenby AN. POLYCHORD: next-generation nested sampling. "Mon. Not. Roy. Astron. Soc.". 2015;453(4):4384–4398. doi:10.1093/mnras/stv1911.

[6] Handley W. fgivenx: Functional Posterior Plotter. The Journal of Open Source Software. 2018;3(28). doi:10.21105/joss.00849.

[7] Hazra DK. ELiXSIR – Extended, zone Linked IX-compartmental SIR model: a code to simulate COVID19 infection; 2021. Available from: https://gitlab.com/dhirajhazra/eSIR_INDIA.

[8] Lewis A. GetDist: a Python package for analysing Monte Carlo samples; 2019. Available from: https://arxiv.org/abs/1910.13970.

[9] Home - Directorate of Economics and Statistics;. Available from: https://des.karnataka.gov.in/english.

[10] Suraksha P. Nearly 30% Covid-19 prevalence rate in Bengaluru: Serosurvey | Deccan Herald; 2020. Available from: https://www.deccanherald.com/city/top-bengaluru-stories/nearly-30-covid-19-prevalence-rate-in-bengaluru-serosurvey-912640.html.

[11] How much do tests for Covid-19 cost in India? A state-wise breakup; 2020. Available from: https://indianexpress.com/article/india/covid-19-test-prices-rates-india-6896237/.

[12] Nandakumar P. Karnataka sero survey: 27% of population may have been infected by COVID-19; 2020. Available from: https://www.theweek.in/news/india/2020/11/04/karnataka-sero-survey-27-of-population-may-have-been-infected-by-covid-19.html.

[13] Chennai district; 2021. Available from: https://en.wikipedia.org/w/index.php?title=Chennai_district&oldid=1036488777.

[14] Selvaraju S, Kumar MS, Thangaraj JWV, Bhatnagar T, Saravanakumar V, Kumar CPG, et al. Population-Based Serosurvey for Severe Acute Respiratory Syndrome Coronavirus 2 Transmission, Chennai, India - Volume 27, Number 2—February 2021 - Emerging Infectious Diseases journal - CDC. Emerging infectious diseases. 2021;doi:10.3201/eid2702.203938.

[15] Malani A, Ramachandran S, Tandel V, Parasa R, Sudharshini S, Prakash V, et al. SARS-CoV-2 Seroprevalence in Tamil Nadu in October-November 2020. medRxiv. 2021; p. 2021.02.03.21250949. doi:10.1101/2021.02.03.21250949.

[16] Sharma N, Sharma P, Basu S, Saxena S, Chawla R, Dushyant K, et al. The seroprevalence and trends of SARS-CoV-2 in Delhi, India: A repeated population-based seroepidemiological study. medRxiv. 2020; p. 2020.12.13.20248123. doi:10.1101/2020.12.13.20248123.

[17] Population projections for India and states 2011–2036;. Available from: https://nhm.gov.in/New_Updates_2018/Report_Population_Projection_2019.pdf.

[18] Rukmini S. COVID-19: 'Indian Exceptionalism' May Not Explain Low Mortality; 2020. Available from: https://www.indiaspend.com/covid-19-indian-exceptionalism-may-not-explain-low-mortality/.

[19] Maharashtra Population 2021;. Available from: https://www.indiacensus.net/states/maharashtra.

[20] Ghose A, Bhattacharya S, Karthikeyan AS, Kudale A, Monteiro JM, Joshi A, et al. Community prevalence of antibodies to SARS-CoV-2 and correlates of protective immunity in five localities in an Indian metropolitan city. medRxiv. 2020; p. 2020.11.17.20228155. doi:10.1101/2020.11.17.20228155.
